# Biological Profiling of Semisynthetic C19-Functionalized Ferruginol and Sugiol Analogues

**DOI:** 10.3390/antibiotics10020184

**Published:** 2021-02-12

**Authors:** Miguel A. González-Cardenete, Fatima Rivas, Rachel Basset, Marco Stadler, Steffen Hering, José M. Padrón, Ramón J. Zaragozá, María Auxiliadora Dea-Ayuela

**Affiliations:** 1 Instituto de Tecnología Química (UPV-CSIC), Universitat Politècnica de València-Consejo Superior de Investigaciones Científicas, Avda. de los Naranjos s/n, 46022 Valencia, Spain; 2 Department of Chemical Biology and Therapeutics, St. Jude Children’s Research Hospital, Memphis, TN 38105, USA; fatima.rivas@stjude.org (F.R.); rachel.bassett@stjude.org (R.B.); 3 Department of Pharmacology and Toxicology, University of Vienna, Althanstrasse 14, A-1090 Vienna, Austria; marco.stadler@univie.ac.at (M.S.); steffen.hering@univie.ac.at (S.H.); 4 BioLab, Instituto Universitario de Bio-Orgánica “Antonio González” (IUBO-AG), Universidad de La Laguna, C/Astrofísico Francisco Sanchez 2, 38200 La Laguna, Spain; jmpadron@ull.es; 5 Departamento de Química Orgánica, Universidad de Valencia, Dr. Moliner 50, 46100 Burjassot, Spain; ramon.j.zaragoza@uv.es; 6 Departamento de Farmacia, Facultad Ciencias de la Salud, Universidad CEU Cardenal Herrera, C/Ramón y Cajal s/n, 46115 Alfara del Patriarca, Spain

**Keywords:** abietane, callitrisic acid, diterpenoid, antiproliferative activity, GABA_A_ receptor modulators, antimalarial activity

## Abstract

The abietane-type diterpenoids are significant bioactive compounds exhibiting a varied range of pharmacological properties. In this study, the first synthesis and biological investigation of the new abietane-diterpenoid (+)-4-epi-liquiditerpenoid acid (8a) together with several of its analogs are reported. The compounds were generated from the readily available methyl callitrisate (7), which was obtained from callitrisic acid present in Moroccan Sandarac resin. A biological evaluation was conducted to determine the effects of the different functional groups present in these molecules, providing basic structure–activity relationship (SAR) elements. In particular, the ferruginol and sugiol analogs compounds **10**–**16** were characterized by the presence of a phenol moiety, higher oxidization states at C-7 (ketone), and the hydroxyl, methyl ester or free carboxylic acid at C19. The biological profiling of these compounds was investigated against a panel of six human solid tumor cell lines (HBL-100, A549, HeLa, T-47D, SW1573 and WiDr), four parasitic *Leishmania* species (*L. donovani*, *L. infantum*, *L. guyanensis* and *L. amazonensis*) and two malaria strains (3D7 and K1). Furthermore, the capacity of the compounds to modulate gamma-aminobutyric acid type A (GABA_A_) receptors (α_1_β_2_γ_2s_) is also described. A comparison of the biological results with those previously reported of the corresponding C18-functionalized analogs was conducted.

## 1. Introduction

Chemical diversity in natural products widely attracts scientific attention to find potential therapeutic agents like anticancer drugs from natural sources. Around 79% of commercial anticancer drugs are either natural products (NPs), directly derived from NPs or inspired in NPs structures [1]. NPs are also sources of novel and selective agents for the treatment of parasitic diseases. Currently, many compounds from plant sources have displayed potential antileishmanial activity; however, none of them have yet undergone clinical trials [2].

**Figure 1:**
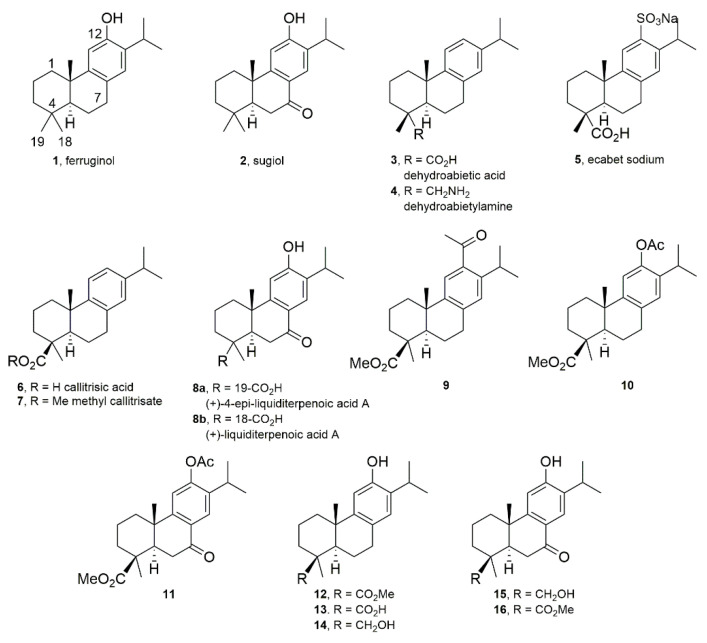
Examples of bioactive abietane diterpenoids 1–7 and tested molecules 8a, and 9–16.

Abietane diterpenoids are naturally occurring metabolites isolated from a large variety of terrestrial plants that show a wide range of promising biological activities [3,4]. These compounds are characterized by a tricyclic ring system. For example, the phenol abietane-type diterpenoid ferruginol (1) (Figure 1) has demonstrated antitumor activity, decreasing non-small cell lung cancer growth after inciting caspase-associated apoptosis. Intraperitoneal administration of ferruginol considerably inhibited the growth of subcutaneous cancer xenograft models [5]. Ferruginol (1) also displays antiproliferative properties in human ovarian cancer and malignant melanoma cells by inhibiting cancer cell migration and inducing apoptosis [6,7], as well as in human thyroid cancer cells [8]. The corresponding C7-oxidized sugiol (2) has shown in vitro cytotoxic activity against human pancreatic and melanoma tumor cell models [9], antitumor activity against EBV-EA activation [10], and in vivo antitumor properties in a prostate DU145 xenograft murine model [11]. Typical semisynthetic bioactive abietane diterpenoids are derived from dehydroabietic acid (3) and dehydroabietylamine (4) (Figure 1), also called leelamine [3].

**Scheme 1:**
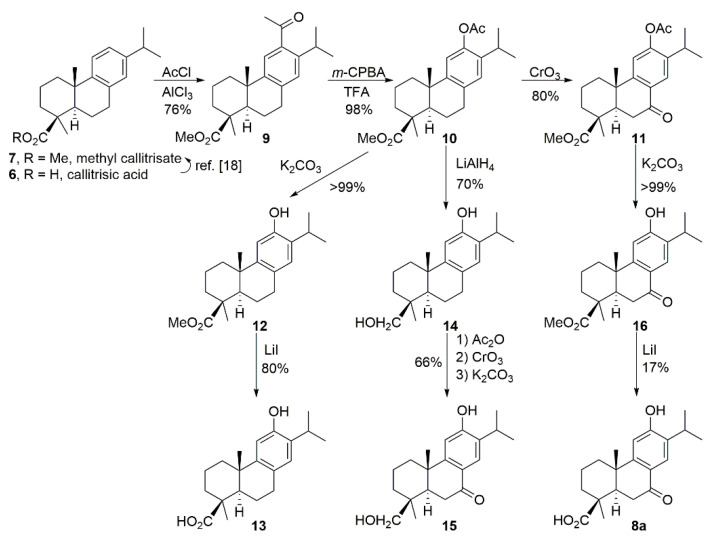
Synthesis of the tested compounds 8a and 9–16.

Dehydroabietic acid (3) displays antileishmanial activity in both promastigotes and amastigotes of *Leishmania amazonensis* [12]. Previously, Moreira and coworkers reported on the antileishmanial activities of dehydroabietic acid derivatives and dehydroabietylamine amides, which were capable of killing *Leishmania donovani* amastigotes [13,14]. Ferruginol (1) has also shown promising antileishmanial activity against *L. donovani* promastigotes [15]. In addition, dehydroabietic acid (3) from *Boswellia thurifera* resin has been reported as a positive gamma-aminobutyric acid type A (GABA_A_) receptor modulator [16]. Recently, we reported on the synthesis and biological activity of (+)-liquiditerpenoic acid A or abietopinoic acid [17]. In that work, we synthesized and studied biologically (antitumor, GABA_A_ modulation and antileishmanial) a series of ferruginol analogs functionalized at C-18 from (–)-abietic acid via methyl dehydroabietate (C-4 epimeric compounds of those depicted in Scheme 1). Despite the important pharmacological properties of abietane diterpenoids, there is only one commercial drug, ecabet, composed of abietane-derived diterpenoids (ecabet sodium (5) (Figure 1) for the treatment of reflux esophagitis and peptic ulcer disease). Thus, this family of natural products offers ample opportunity for further studies due to their promising biological activities. In continuation of our research programs on the synthesis and biological analysis of bioactive terpenoid compounds, the synthesis and biological studies of several ferruginol and sugiol analogs functionalized at C-19 is presented herein. The synthesis commenced with methyl callitrisate (7) obtained from the naturally occurring callitrisic acid (6) present in Sandarac resin from Morocco (Figure 1), following our reported protocol [18].

In the current study, the semisynthesis of (+)-4-epi-liquiditerpenoic acid A (8a) and analogs (9–16) from methyl callitrisate (7) (Scheme 1) was conducted, their evaluation in a panel of six human solid tumors, four *Leishmania* species, inhibition of growth of malaria strains 3D7 and K1, and their modulating activity of GABA_A_ receptor subtype α_1_β_2_γ_2s_ are reported. Our main objective is to identify new drug leads from the abietanes by better understanding their SAR, and we particularly focused on modifying the functional groups at C-7, C-12 and C-19 to compare with the corresponding C18-functionalized equivalents. It should be noted that very few derivatives of callitrisic acid (6) have been biologically evaluated, making these efforts of great value to the field.

## 2. Results and Discussion

### 2.1. Chemistry

The tested compounds **8a** and **9**–**16** were obtained from (+)-methyl callitrisate (7), which was obtained from callitrisic acid (6) extracted from Sandarac resin [18], as shown in Scheme 1. Compounds 8a, 15 and 16 are novel derivatives. The remaining compounds have either been isolated from natural sources or synthesized by other methods.

Based on previous studies during the synthesis of (–)-sugikurojin A [19], compounds 9 and later 10 were prepared by Friedel–Crafts and Baeyer–Villiger reactions to generate the C-12 phenolic moiety giving the key intermediate 10 (Scheme 1) in 76% yield for the first step and 98% yield for the second step. Compound 10 is useful to provide a series of diverse analogs and 4-epi-liquiditerpenoic acid A (8a). First, benzylic oxidation at the C-7 position gave ketone 11, which after methanolysis afforded sugiol analog 16. The nucleophilic cleavage of methyl ester 16 provided the (+)-4-epi-liquiditerpenoic acid A (8a) in 10% overall yield for the five steps. Additional functional group handling of intermediate 10 led after deacetylation to phenol 12, which was then reacted with LiI to afford lambertic acid (13). Another approach gave directly 19-hydroxyferruginol (14) via reduction of 10. Compound 14 was next acetylated, oxidized at C-7, and hydrolyzed to give the new compound 19-hydroxysugiol (15) (Scheme 1). All the obtained compounds presented high purity (>95%) by elemental analysis and were synthesized stereoselectively since the stereochemistry at C-4 is fixed in the starting material. Experimental procedures for the synthesis of compounds **8a**, **12**–**16** and their corresponding copies of NMR (^1^H, ^13^C and DEPT) spectra are provided in the Supplementary Materials.

### 2.2. Biology

#### 2.2.1. Antiparasitic Activity

Compounds **8a** and **9**–**16** (Scheme 1) were studied for antileishmanial activity in four *Leishmania* species (*L. donovani*, *L*. *infantum*, *L. guyanensis* and *L. amazonensis*), and for cytotoxicity against J774 macrophages, according to established procedures [20]. The results on promastigotes are displayed in Table 1, while those on amastigotes of *L. infantum* and *L. amazonensis* are summarized in Table 2. The antimalarial and cytotoxic activities of ferruginol analogs 8a and 9–16 (Scheme 1) were also evaluated against malaria strains 3D7 (chloroquine-sensitive) and K1 (chloroquine-resistant), following a previously reported assay [21]. The effects of the compounds on *Plasmodium falciparum* were calculated as the minimum drug concentration (EC_50_) required to inhibit parasite growth by more than 50% for 72 h incubation period with test compounds. The compounds were additionally studied in a panel of mammalian cell lines to determine cytotoxicity. Only those molecules with a relevant selectivity index (SI) are potential lead candidates. The results are displayed in Table 3.

As it is shown in Table 1, compounds **8a**, **9**, **13** and **16** displayed important activity without significant cytotoxicity on J774 macrophage cells (CC_50_ > 200 μM), being compound 8a the most potent for *L. guyanensis* (IC_50_ = 3.0 μM, SI > 67). Compound 10, the key intermediate, resulted in being the most active molecule; however, it presented some cytotoxicity on J774 cells. The remaining ferruginol analogs, compounds **11**–**12** and **14**–**15**, also presented cytotoxicity and were not further studied. It is noteworthy that sugiol analog 16 exhibited better activity against *L. amazonensis* (IC_50_ = 8.5 μM, SI > 23.5) and *L. guyanensis* (IC_50_ = 5.6 μM, SI > 35.7) parasites compared to the drug miltefosine, used as control. It also displayed a better selectivity index (SI = CC_50_/IC_50_) in those *Leishmania* spp. In the mammalian hosts, *Leishmania* parasites occur as intracellular amastigotes inside phagolysosomes of macrophages.

Compounds **8a**, **9**, **13** and **16** are the most active agents in the antipromastigote assay (SI from >2 to >67) and could be good candidates for further research against the clinically relevant *Leishmania* amastigote forms. Therefore, these ferruginol analogs were tested against *L. infantum* and *L. amazonensis* amastigotes (Table 2). When testing on *L. infantum* amastigotes, the antileishmanial activity of the tested compounds was comparable to that of extracellular forms, except for compound **8a**, which demonstrated superior potency. Interestingly, *L. amazonensis* amastigotes display higher sensitivity to compounds **8a** and **13**. Compound **13** was the most potent against *L. amazonensis* amastigotes (IC_50_ = 8.8 μM). Some authors have claimed that a compound should have a selectivity index (SI) value >20 to be considered as a potential leishmanicidal agent [22]. This requirement is satisfied by compound **13** (SI > 24.7) against *L. amazonensis*.

Some structure–activity relationship is deduced in the non-cytotoxic (CC_50_ > 200 μM) ferruginol analogs. For example, the introduction of a carbonyl group at C-7 in synthetic lambertic acid (13) led to a less active derivative, compound **8a**. If the latter is esterified as methyl ester at C19 leads to compound **16** with an improved pharmacological profile.

On comparing these antileishmanial results with those of the corresponding C18-functionalized equivalents (see ref. [17]), some structure–activity relationships could be established. Overall, it can be deduced that the C19-functionalized derivatives are less cytotoxic on J774 cells. It is interesting to note that 4-epi-liquiditerpenoic acid A (8a) displayed activity in the tested promastigote *Leishmania* spp. strains, particularly, remarkable activity was observed against *L. guyanensis* while (+)-liquiditerpenoic acid A (8b, Figure 1) was inactive [17].

While no C19-functionalized derivative showed better potency than the reference drug (miltefosine) for *L. donovani*, it was gratifying to observe improved activity for the C18-congeners. The acetyl derivative 9 was less active, in general than the corresponding C4-epimer in the promastigote stage, but of similar potency in the amastigote forms. Furthermore, after comparing antipromastigote activity of synthetic lambertic acid (13) (IC_50_ values from 12.8 to 51.5 μM) with the corresponding 12-hydroxydehydroabietic acid (IC_50_ values from 43.6 to 71.4 μM, ref. [17]), both superior potency and SI (2.5-fold less cytotoxic) were recorded. It is also important to highlight that the methyl ester of liquiditerpenoic acid A, under the experimental conditions, had solubility problems and was not tested. Herein, we disclose that its corresponding C-19 methyl ester, compound 16, showed superior activity in both promastigote and amastigote stages over the current standard treatment with miltefosine. The resultant data also indicates that the presence of either an acetate group at C12 or a hydroxyl group at C19 leads to more cytotoxic compounds. Briefly, it seems to be essential for antileishmanial activity C19-carboxylic acid or methyl ester along with a C12-hydroxyl group as well as an acetyl group at C12 might lead to active compounds.

The inhibitory values against *P. falciparum* strains ranged from 2.1 to 12.8 μM, with cytotoxicity properties >25 μM (Table 3). Unfortunately, no tested compound improved the values for the parent ferruginol (1) [23]. In general, the presence of either an acetate group at C12 or a hydroxyl group at C19 decreased the biological activity of these compounds (**10**, **11** and **14**, **15**).

#### 2.2.2. Antiproliferative Activity

The ability of compounds 8a and 9–16 to inhibit the proliferation of the human solid tumor cell lines HBL-100 (breast), A549 (lung), HeLa (cervix), T-47D (breast), SW1573 (lung), and WiDr (colon) was evaluated in vitro by using the established sulforhodamine B (SRB) [24]. The results are shown in Table 4 (GI_50_). The anticancer drugs cisplatin and etoposide were used for reference. Most of the molecules were active (GI_50_ < 25 μM) in the solid tumor cells with higher activity than parent ferruginol (1) and sugiol (2) as reported by Li et al. [25] for A549 cell line and compounds 10–12 better than the starting methyl callitrisate (7) comparing with previous results [26]. The target molecule, (+)-4-epi-liquiditerpenoic acid (8a), showed better activity than sugiol (2) for the A549 cell line. The most potent compound, in general for the C19-functionalized series, was compound 14 (19-hydroxyferruginol), characterized by a hydroxyl group at C12 and a hydroxymethyl group at C19. Its activity (1.8 to 13.0 μM ) was comparable to etoposide except for the HBL-100 cell line. The moderate cytotoxic activity of 19-hydroxyferruginol (14) for the A549 cell line has been reported to be IC_50_ = 14 μM after measuring by the MTT (3-(4,5-dimethylthiazol-2-yl)-2,5-diphenyl tetrazolium bromide) method [27]. Compounds 10–12 and 14 were more potent than cisplatin and etoposide for the more resistant cell lines WiDr and T-47D, demonstrating the potential for further mechanistic studies. Compounds 10–11 were also more potent against the A549 cell line than the drug cisplatin. One clear SAR that can be established is that oxidation at C-7 results in less potent molecules for esters and alcohols (Table 4, compounds **12** and **14** vs. **16** and **15**, respectively). On comparing with the corresponding C18-functionalized equivalents [17], in general, the C19-functionalized series is more potent. In particular, the corresponding C18-functionalized analogs of compounds 8a, 13, 14 and 16 were less potent for all tested cell lines. Specifically, compound 11 (for HBL-100) and compound 12 (for T-47D and WiDr) and compound 14 (for A549 and HeLa) were the most potent compounds of all C18- and C19-functionalized analogs. Briefly, it seems to be key for important antiproliferative activity in an acetate group at C12 and a C19-hydroxymethyl group. This combination is interesting for designing new molecules.

#### 2.2.3. GABA_A_ Receptor Modulating Activity

The compounds 8a and 9–16 were tested for their properties on the GABA_A_ receptor subtype (α_1_β_2_γ_2S_) by means of the two-microelectrode voltage-clamp technique in *Xenopus laevis* oocytes [17,26]. All molecules were tested at concentrations of 10, 100 μM and compared with dehydroabietic acid (DHA) (3) as a reference [16]. We considered this study interesting since we could obtain some basic SAR on changing the functionalization at C-19 along with the oxygenated moiety at C-12, in comparison to dehydroabietic (3) and callitrisic (6) acids. The results are displayed in Table 5. 19-Hydroxyferruginol (14) showed considerable potentiation (I_GABA_ = 507% ± 102%) of GABA-evoked currents at 100 μM and introduction of a ketone at C-7, compound 15, led to less potent analog (I_GABA_ = 238% ± 28%). On comparing with the corresponding C18-congeners, the most potent was still 19-hydroxyferruginol (14), and clearly, the change in stereochemistry at C4 reduced activity. Interestingly, the contrary happened for compounds 12 and 16; both methyl esters, which corresponding to C18-functionalized analogs (12b and 16b), were more potent compounds. Briefly, it seems to be essential for GABA_A_ potentiation of a C19-hydroxymethyl group in these series of compounds along with a C12-hydroxyl group.

## 3. Materials and Methods

### 3.1. General Experimental Procedures

The general details and the extraction of the starting material methyl callitrisate (7) from Moroccan sandarac resin were reported previously [18].

### 3.2. Chemistry

*Materials.* Key intermediates 10 and 11 were obtained according to conditions reported by us [19]. All compounds prepared in this work display spectroscopic data in agreement with the proposed structures. The purity of all final compounds was 95% or higher.

### 3.3. Biology

#### 3.3.1. Antileishmanial Activity

The parasites, culture procedure and the activity of functionalized abietane analogs on *Leishmania* promastigotes were performed according to the method previously reported by us [28], while the intracellular amastigote assay was performed on *L*. *amazonensis* and *L*. *infantum* according to the fluorometric test [29]. The J774 cell culture and the cytotoxicity assay was carried out according to our previously described method [30].

#### 3.3.2. Antiproliferative Activity

Cells, cultures and the activity test of abietane analogs followed reported methods [26]. The SRB assay was performed as previously described [24], and the antiproliferative activity was given as GI_50_ values, which were calculated according to NCI formulas [24].

#### 3.3.3. Expression of GABA_A_ Receptors in Xenopus Laevis Oocytes

The experiments were carried out according to procedures formerly described [16,26,31].

#### 3.3.4. Antimalarial Activity

Assays were performed by using previously described DNA stain-based assay and methods, including cytotoxicity assays [21,23].

## 4. Conclusions

To sum up, we have carried out the first chemical synthesis of the abietane (+)-4-epi-liquiditerpenoic acid A (8a). It was prepared in a short synthetic sequence of five steps in 10% overall yield from methyl callitrisate (7). Two parallel routes led to several oxidized analogs, such as naturally occurring lambertic acid (13) or 19-hydroxyferruginol (14). Their accessibility permitted biological profiling of several analogs based on the parent pharmacophore structure of ferruginol (1), that is, 12-hydroxy-8,11,13-abietatriene. This work expands the knowledge on a group of bioactive abietanes and enables us to access additional analogs of these diterpenoids using similar procedures. However, issues of cytotoxicity with respect to antileishmanial activity need to be further studied [32]. Our results demonstrate that these abietane-diterpenoid analogs are sources of promising bioactive molecules with interesting pharmacological properties.

## Figures and Tables

**Table 1:** Antileishmanial and cytotoxicity (IC_50_ in μM) of C19-functionalized ferruginol and sugiol analogs on in vitro promastigote assay.

Compound	*L. infantum*		*L. donovani*		*L. amazonensis*		*L. guyanensis*		Macrophages J774
	IC_50_^a^ ± SD	SI ^b^	IC_50_ ± SD	SI	IC_50_ ± SD	SI	IC_50_ ± SD	SI	CC_50_
**1** ^d^	-	-	12.2	>1.4	-	-	-	-	>16.6 (Vero)
**8a**	100.3 ± 20.0	>2.0	84.4 ± 31.6	>2.4	94.5 ± 6.5	>2.1	3.0 ± 0.9	>67.0	>200
**8b** ^e^	NA ^f^		NA ^f^		NA ^f^		NA ^f^		219.4 ± 15.0
**9**	20.3 ± 4.6	>10.0	8.9 ± 2.0	>22.0	14.1 ± 1.4	>14.0	18.7 ± 0.8	>10.7	>200
**9b** ^e^	2.5 ± 0.6	51.8	14.8 ± 0.9	8.8	11.6 ± 0.6	11.2	14.2 ± 0.4	9.1	129.6 ± 8.9
**10**	3.6 ± 0.6	>8.4	3.9 ± 0.1	>7.7	7.4 ± 0.6	>4.1	ND ^g^	-	30.2 ± 2.0
**10b** ^e^	1.3 ± 0.1	20.4	8.1 ± 0.7	3.3	3.2 ± 0.3	8.2	5.5 ± 0.7	4.8	26.5 ± 2.7
**11**	ND ^g^	-	ND ^g^	-	ND ^g^	-	ND ^g^	-	45.8 ± 5.6
**11b** ^e^	0.7 ± 0.2	25.4	4.4 ± 0.5	4.1	0.65 ± 0.03	27.4	1.3 ± 0.1	13.6	17.8 ± 2.8
**12**	ND ^g^	-	ND ^g^	-	ND ^g^	-	ND ^g^	-	65.4 ± 7.0
**12b** ^e^	5.0 ± 0.3	6.8	9.21 ± 0.06	3.7	3.9 ± 0.1	8.7	5.9 ± 1.1	5.8	34.2 ± 3.1
**13**	29.1 ± 1.5	7.5	12.8 ± 2.8	17.0	51.5 ± 3.2	4.2	17.8 ± 1.2	12.2	217.4 ± 34.0
**13b** ^e^	43.6 ± 3.4	2.0	58.1 ± 0.7	1.5	54.1 ± 6.5	1.6	71.4 ± 1.5	1.2	85.8 ± 28.8
**14**	ND ^g^	-	ND ^g^	-	ND ^g^	-	ND ^g^	-	51.9 ± 5.9
**14b** ^e^	0.7 ± 0.1	33.4	13.9 ± 0.1	1.7	7.7 ± 0.8	3.0	12.5 ± 2.2	1.9	23.4 ± 4.6
**15**	ND ^g^	-	ND ^g^	-	ND ^g^	-	ND ^g^	-	50.1 ± 2.4
**15b** ^e^	9.7 ± 0.8	1.5	19.7 ± 0.4	0.7	10.2 ± 0.6	1.4	16.5 ± 0.7	0.9	14.4 ± 2.8
**16**	10.1 ± 1.4	>19.8	11.8 ± 0.8	>16.9	8.5 ± 0.3	>23.5	5.6 ± 0.6	>35.7	>200
**16b** ^e^	ND ^g^		ND ^g^		ND ^g^		ND ^g^		153.9 ± 49.3
Miltefosine	3.4 ± 0.6	40.1	0.15 ± 0.02	909	47.7 ± 5.0	2.9	18.2 ± 0.6	7.5	136.4 ± 1.4

^a^ IC_50_, concentration of the compound that gave a 50% reduction in parasites; SD: standard deviation; ^b^ selectivity index, SI = CC_50_/IC_50._^c^ CC_50_ (μM), concentration of the compound that gave a 50% reduction of cell viability in treated culture cells with respect to untreated ones; ^d^ values taken from reference [15]; ^e^ refers to C18-congeners. Values taken from reference [17]; ^f^ NA, no activity; ^g^ ND, not determined.

**Table 2:** Antileishmanial and cytotoxicity (IC_50_ in μM) of C19-functionalized ferruginol and sugiol analogs on amastigotes.

Compound	*L. infantum*		*L. amazonensis*		*Macrophages* J774
	IC_50_^a^ ± SD	SI ^b^	IC_50_ ± SD	SI	CC_50_^c^
**8a**	20.1 ± 2.0	>9.9	21.4 ± 2.3	>9.3	>200
**9**	22.7 ± 5.9	>8.8	30.3 ± 1.2	>6.6	>200
**9b** ^d^	37.2 ± 2.4	3.5	31.4 ± 6.2	4.1	129.6 ± 8.9
**13**	19.2 ± 3.8	>11.3	8.8 ± 1.6	>24.7	217.4 ± 34.0
**16**	16.5 ± 4.6	>12.1	16.1 ± 2.0	>12.4	>200
Miltefosine	58.1 ± 4.4	2.3	16.5 ± 1.9	8.2	136.4 ± 1.4

^a^ IC_50_, concentration of the compound that gave a 50% reduction in parasites; SD: standard deviation. ^b^ Selectivity index, SI = CC_50_/IC_50._^c^ CC_50_ (μM), concentration of the compound that gave a 50% reduction of cell viability in treated culture cells with respect to untreated ones; ^d^ refers to C18-congener. Values taken from reference [17].

**Table 3:** Antiplasmodial activity and cytotoxicity (EC_50_-μM) of ferruginol and sugiol analogs 8a, 9–16 on *P. falciparum* 3D7 strain, K1 strain, and HepG2, RAJI, BJ, and HEK293 cell lines.

Compound	*P. falciparum* ^a^				Cell Lines ^b^			
	3D7		K1		HepG2	RAJI	BJ	HEK293
	EC_50_^c^	SI ^d^	EC_50_^c^	SI ^d^	EC_50_^c^	EC_50_^c^	EC_50_^c^	EC_50_^c^
**1** ^e^	2.47	4.6	1.33	8.6	>25	11.49	19.57	>25
**8a**	>15	n.c.	>15	n.c.	>25	>25	>25	>25
**9**	12.8	1.9	12.6	2.0	>25	>25	>25	>25
**10**	>15	n.c.	3.1	8.1	>25	>25	>25	>25
**11**	7.1	3.5	5.7	4.4	>25	>25	>25	>25
**12**	4.7	5.3	2.1	11.9	>25	>25	>25	>25
**13**	>15	n.c.	>15	n.c.	>25	>25	>25	>25
**14**	>15	n.c.	11.6	2.2	>25	>25	>25	>25
**15**	>15	n.c.	9.4	2.6	>25	>25	>25	>25
**16**	4.8	5.2	3.9	6.4	>25	>25	>25	>25
Chloroquine	0.023	n.c.	0.69	n.c.	>25	>25	>25	>25
Gambogic acid	-	-	-	-	>25	0.24	0.21	0.21
Staurosporine	-	-	-	-	<0.2	<0.2	<0.2	<0.2

^a^ 3D7 (chloroquine-sensitive) and K1 (chloroquine-resistant); ^b^ BJ, normal human foreskin fibroblast; HEPG2, human liver carcinoma cell line; HEK293, human embryonic kidney cell line; RAJI, Burkitt lymphoma cell line; ^c^ EC50 (μM): concentration corresponding to 50% growth inhibition of the parasite or cells; ^d^ selectivity index (SI): EC_50_ lower values of cytotoxic activity/EC_50_ values of antimalarial activity. N.C.: not calculated; ^e^ values taken from reference [23].

**Table 4:** Antiproliferative activity (GI_50_) of 4-epi-liquiditerpenoic acid (8a), and analogs 9–16 against some human solid tumor cells ^a^.

Compound	Cell Line (Origin)					
	A549 (Lung)	HBL-100 (Breast)	HeLa (Cervix)	SW1573 (Lung)	T-47D (Breast)	WiDr (Colon)
**1** ^b^	30.7 ± 5.2	-	-	-	-	-
**2** ^b^	79.8 ± 2.2	-	-	-	-	-
**7** ^c^	10.0 ± 1.9	14.0 ± 5.0	16.0 ± 2.5	17.0 ± 2.2	8.8 ± 2.7	6.4 ± 2.1
**8a**	12.0 ± 2.9	39.0 ± 14.0	12.0 ± 3.8	41.0 ± 17.0	31.0 ± 12.0	33.0 ± 15.0
**8b** ^d^	>100	>100	85.0 ± 21.0	>100	>100	>100
**9**	15.0 ± 7.0	14.0 ± 2.1	15.0 ± 4.1	25.0 ± 11.0	14.0 ± 1.0	15.0 ± 2.1
**9b** ^d^	18.0 ± 2.3	15.0 ± 1.2	11.0 ± 1.0	11.0 ± 4.6	10.0 ± 1.5	17.0 ± 2.4
**10**	6.5 ± 2.7	11.0 ± 2.3	7.0 ± 1.7	18.0 ± 3.4	4.2 ± 1.5	4.5 ± 1.4
**10b** ^d^	11.0 ± 1.8	15.0 ± 3.9	4.4 ± 1.1	12.0 ± 3.2	14.0 ± 2.9	16.0 ± 2.5
**11**	3.1 ± 0.9	7.4 ± 0.7	5.6 ± 2.9	17.0 ± 7.5	6.1 ± 1.9	5.2 ± 3.1
**11b** ^d^	14.0 ± 0.4	16.0 ± 1.5	13.0 ± 2.3	17.0 ± 1.9	20.0 ± 2.9	19.0 ± 1.7
**12**	2.3 ± 1.5	11.0 ± 3.0	5.1 ± 1.9	12.0 ± 4.1	4.2 ± 1.3	3.7 ± 1.9
**12b** ^d^	9.4 ± 2.6	9.7 ± 2.7	5.8 ± 0.5	11.0 ± 2.0	19.0 ± 3.8	23.0 ± 4.4
**13**	17.0 ± 6.1	34.0 ± 4.2	29.0 ± 13.0	32.0 ± 8.9	41.0 ± 8.4	59.0 ± 7.3
**13b** ^d^	70.0 ± 6.3	>100	96.0 ± 5.5	93.0 ± 9.3	>100	>100
**14**	1.8 ± 0.4	13.0 ± 1.6	3.1 ± 0.9	13.0 ± 1.3	12.0 ± 1.8	13.0 ± 3.7
**14b** ^d^	12.0 ± 1.9	16.0 ± 2.0	12.0 ± 1.8	15.0 ± 2.8	16.0 ± 0.9	17.0 ± 1.3
**15**	10.0 ± 0.3	17.0 ± 1.7	15.0 ± 1.1	19.0 ± 3.3	19.0 ± 1.1	17.0 ± 5.0
**15b** ^d^	19.0 ± 3.3	16.0 ± 2.0	16.0 ± 2.4	23.0 ± 0.7	20.0 ± 2.7	19.0 ± 2.1
**16**	11.0 ± 6.9	65.0 ± 4.9	18.0 ± 1.7	>100.0	47.0 ± 25.0	6.1 ± 4.2
**16b** ^d^	>100	>100	>100	>100	>100	>100
etoposide	1.5 ± 0.3	1.4 ± 0.1	3.3 ± 1.6	15.0 ± 1.5	22.0 ± 5.5	23.0 ± 3.1
cisplatin	4.9 ± 0.2	1.9 ± 0.2	1.8 ± 0.5	2.7 ± 0.4	17.0 ± 3.3	23.0 ± 4.3

^a^ Values are given in μM and represent the mean ± standard deviation of at least two independent experiments; ^b^ values taken from reference [25]; ^c^ values taken from reference [26]; ^d^ refers to C18-congeners. Values taken from reference [17].

**Table 5:** Potentiation of I_GABA_ in α1β2γ2S receptors by compounds 8a, and 9–16.

Compound	I_GABA_ Potentiation of 10 µM (%)	I_GABA_ Potentiation of 100 µM (%)
Dehydroabietic acid (3)	192 ± 10	789 ± 82
Callitrisic acid (6) ^a^	14 ± 5	269 ± 24
**8a**	−8 ± 1	−8 ± 3
**8b** ^b^	−2.2 ± 4.4	−21.1 ± 7.1
**9**	-	-
**9b** ^b^	2.7 ± 0.9	−2.9 ± 5.1
**10**	-	-
**10b** ^b^	5.1 ± 2.1	26.3 ± 8.9
**11**	1 ± 3	75 ± 4
**11b** ^b^	−1.0 ± 2.8	46.7 ± 8.3
**12**	1 ± 3	65 ± 6
**12b** ^b^	23.7 ± 9.6	378.0 ± 65.6
**13**	3 ± 3	71 ± 23
**13b** ^b^	8.9 ± 2.3	12.1 ± 2.9
**14**	64 ± 13	507 ± 102
**14b** ^b^	29.3 ± 10.6	154.1 ± 34.2
**15**	30 ± 14	238 ± 28
**15b** ^b^	4.5 ± 5.8	−30.3 ± 9.5
**16**	5 ± 4	104 ± 26
**16b** ^b^	37.4 ± 12.7	242.4 ± 46.0

^a^ Values taken from reference [26]; ^b^ refers to C18-congeners. Values taken from reference [17].

## Data Availability

Not applicable.

## Supplementary Materials

The following are available online at https://www.mdpi.com/2079-6382/10/2/184/s1. Experimental procedures for the synthesis of compounds 8a, 12–16 and their corresponding copies of NMR spectra.
