# Supplementary material for: Biological Profiling of Semisynthetic C19-Functionalized Ferruginol and Sugiol Analogues
Source: Antibiotics (Basel). 2021 Feb 12;10(2):184. doi: 10.3390/antibiotics10020184 (PMC7918733; doi:10.3390/antibiotics10020184)
Supplement: Supplementary file 1 [file antibiotics-10-00184-s001.pdf]

Supplementary Material

Biological Profiling of Semisynthetic C19-Functionalized Ferruginol and Sugiol Analogues

Miguel A. González-Cardenete[1,*], *Fatima Rivas*[2], *Rachel Bassett*[2], *Marco Stadler*[3], *Steffen Hering*[3], *José M. Padrón*[4], *Ramón J. Zaragozá*[5], *and M. Auxiliadora Dea-Ayuela*[6,*]

[1] Instituto de Tecnología Química (UPV-CSIC), Universitat Politècnica de València-Consejo Superior de Investigaciones Científicas, Avda. de los Naranjos s/n, 46022 Valencia, Spain

[2] Department of Chemical Biology and Therapeutics, St. Jude Children's Research Hospital, Memphis, TN 38105, USA

[3] Department of Pharmacology and Toxicology, University of Vienna, Althanstrasse 14, A-1090 Vienna, Austria

[4] BioLab, Instituto Universitario de Bio-Orgánica "Antonio González" (IUBO-AG), Centro de Investigaciones Biomédicas de Canarias (CIBICAN), Universidad de La Laguna, C/Astrofísico Francisco Sanchez 2, La Laguna 38200, Tenerife, Spain

[5] Departamento de Química Orgánica, Universidad de Valencia, Dr. Moliner 50, 46100 Burjassot, Valencia, Spain

[6] Departamento de Farmacia, Facultad Ciencias de la Salud. Universidad CEU Cardenal Herrera, C/ Ramón y Cajal s/n, 46115 Alfara del Patriarca (Valencia), Spain.

Number of pages: 25

Number of spectra: 18 (^{1}H, ^{13}C, DEPT135)

General Experimental Procedures

The melting points were measured with a Büchi 535 apparatus and are uncorrected. Optical rotations were measured using a 5 cm cell in a Schmidt-Haensch Polartronic-D polarimeter. NMR spectra were recorded on a 300 MHz spectrometer (^{1}H: 300 MHz, ^{13}C: 75 MHz) and referenced to the solvent peak at 7.26 ppm (^{1}H) and 77.00 ppm (^{13}C) for $CDCl_3$ and 3.31 ppm (^{1}H) and 49.00 ppm (^{13}C) for methanol-d4. All spectra were recorded in $CDCl_3$ as solvent unless otherwise stated. Complete assignments of ^{13}C NMR multiplicities were made on the basis of DEPT experiments, while the ^{13}C spectra were took decoupled. *J* values are given in Hz. MS data were acquired on a QTOF spectrometer. Elemental analysis was performed in a EuroEA 3000 elemental analyzer. Reactions were monitored by TLC using Merck silica gel 60 F-254 in 0.25 mm-thick plates. Compounds on TLC plates were detected under UV light at 254 nm and visualized by immersion in a 10% sulfuric acid solution and heating with a heat gun. Purifications were performed by flash chromatography on Merck silica gel (230-400 mesh). Commercial reagent grade solvents and chemicals were used as purchased unless otherwise noted. Combined organic extracts were washed with brine, dried over anhydrous Na_2SO_4, filtered, and concentrated under reduced pressure.

Synthesis

Methyl 12-hydroxy-abieta-8,11,13-trien-19-oate (12, methyl 12-hydroxycallitrisate). The acetate **10** (327 mg, 0.87 mmol) was dissolved in absolute MeOH (12 mL) and K_2CO_3 (600 mg, 4.34 mmol) was added in portions. The reaction mixture was stirred for 1 h at rt and then diluted with H_2O (60 mL) and extracted with diethyl ether (3 × 20 mL). The combined organic extracts were dried and concentrated. The resulting residue was chromatographed on silica eluting with *n*-hexane-EtOAc (8:2) to give 287 mg (>99%) of phenol **12** as a white solid: mp 182-184 ºC; $[\alpha]^{20}_{D}$ +82 (c 0.7, $CHCl_3$)(lit.,[1] +72.6 (c 0.6, $CHCl_3$)). ^{1}H NMR (300 MHz) δ 6.83 (1H, s), 6.63 (1H, s), 4.60 (1H, s), 3.66 (3H, s), 3.11 (1H, sept., *J* = 6), 2.86-2.65 (2H, m), 2.29-2.24 (1H, m), 2.17-2.13 (2H, m), 2.00-1.91 (2H, m), 1.64-1.58 (1H, m), 1.54-1.49 (1H, m), 1.42-1.35 (1H, m), 1.26 (3H, s), 1.24 (3H, d, *J* = 6), 1.23 (3H, d, *J* = 6), 1.12-1.02 (1H, m), 1.01 (3H, s); ^{13}C NMR (75 MHz) δ_C 178.0 (s), 150.8 (s), 146.5 (s), 131.8 (s), 127.4 (s), 126.7 (d), 112.0 (d), 52.8 (d), 51.2 (q), 44.0

(s), 39.4 (t), 38.1 (s), 37.6 (t), 31.3 (t), 28.5 (q), 26.8 (d), 22.9 (q), 22.7 (q), 22.5 (q), 21.2 (t), 20.0 (t); HRMS (ESI) *m/z* 331.2277 $[M+ H]^+$, calcd for $C_{21}H_{31}O_3$: 331.2273; Anal. calcd. for $C_{21}H_{30}O_3$: C, 76.3; H, 9.2. Found: C, 76.0; H, 9.4.

12-Hydroxyabieta-8,11,13-trien-19-oic acid (13, lambertic acid). A suspension of ester **12** (173 mg, 0.52 mmol) and LiI (480 mg, 3.6 mmol) in 2,4,6-collidine (2.5 mL) was heated at reflux for 2 h under an Argon atmosphere and then cooled to rt. The mixture was treated with ice and 6 N HCl (20 mL) carefully and extracted with DCM (3 × 15 mL). The organic extracts were washed with brine, dried and concentrated. The resulting brown residue was dissolved in DCM and chromatographed on silica eluting with *n*-hexane-EtOAc (1:1) to give 130 mg (80%) of acid **13** as a light pale solid: mp 250-252 ºC (lit.,[2] 252-254 ºC); $[\alpha]^{20}_D$ +113 (c 0.8, EtOH)(lit.,[2] +121.5 (c 0.03, EtOH)). ^1H NMR (300 MHz) δ 6.83 (1H, s), 6.63 (1H, s), 3.76-3.69 (1H, m), 3.11 (1H, sept., *J* = 6), 2.90-2.65 (2H, m), 2.27-2.04 (3H, m), 2.02-1.96 (2H, m), 1.70-1.44 (3H, m), 1.38-1.34 (1H, m), 1.33 (3H, s), 1.24 (3H, d, *J* = 6), 1.23 (3H, d, *J* = 6), 1.13-1.07 (1H, m), 1.11 (3H, s); ^{13}C NMR (75 MHz) δ_C 181.7 (s), 150.8 (s), 146.4 (s), 131.9 (s), 127.4 (s), 126.7 (d), 111.9 (d), 52.7 (d), 43.7 (s), 39.3 (t), 38.3 (s), 37.5 (t), 31.2 (t), 28.7 (q), 26.8 (d), 23.1 (q), 22.7 (q), 22.5 (q), 21.1 (t), 19.9 (t); HRMS (ESI) *m/z* 317.2120 $[M+ H]^+$, calcd for $C_{20}H_{29}O_3$: 317.2117; Anal. calcd. for $C_{20}H_{28}O_3$: C, 75.9; H, 8.9. Found: C, 75.7; H, 8.7.

Abieta-8,11,13-trien-12,19-diol (14, 19-hydroxyferruginol). $LiAlH_4$ (280 mg, 7.4 mmol) was added in portions to a stirred solution of acetate **10** (275 mg, 0.74 mmol) in anhydrous THF (22 mL) at 0 ºC. This mixture was allowed to warm to rt and stirred for 17 h. Then, the reaction mixture was quenched by pouring it carefully into ice-H_2O (40 mL) followed by addition of 2 N HCl (5 mL). Next, the mixture was extracted with ethyl acetate (3 × 15 mL). The combined organic layers were washed with brine, dried and concentrated. The resulting residue was chromatographed on

silica eluting with *n*-hexane-EtOAc (4:6) to give 155 mg (70%) of **14** as a pale solid: mp 162-167 ºC; $[\alpha]^{20}_D$ +54 (c 0.7, $CHCl_3$). The ^1H and ^{13}C NMR data were in agreement with those of the natural product:[3,4] ^1H NMR (300 MHz) δ 6.83 (1H, s), 6.64 (1H, s), 3.88 (1H, d, J = 12), 3.56 (1H, d, J = 12), 3.14 (1H, sept., J = 6), 2.85-2.70 (2H, m), 2.21-2.17 (1H, m), 2.00-1.87 (2H, m), 1.72-1.57 (3H, m), 1.51-1.38 (2H, m), 1.24 (3H, d, J = 6), 1.23 (3H, d, J = 6), 1.16 (3H, s), 1.06 (3H, s); ^{13}C NMR (75 MHz) δ_C 150.9 (s), 148.0 (s), 131.8 (s), 126.6 (s), 126.5 (d), 111.0 (d), 65.2 (t), 51.2 (d), 38.9 (t), 38.6 (s), 37.4 (s), 35.1 (t), 30.2 (t), 26.7 (d), 26.7 (q), 25.6 (q), 22.7 (q), 22.5 (q), 19.3 (t), 19.0 (t); HRMS (ESI) *m/z* 303.2329 $[M+H]^+$, calcd for $C_{20}H_{31}O_2$: 303.2324; Anal. calcd. for $C_{20}H_{30}O_2$: C, 79.4; H, 10.0. Found: C, 79.0; H, 10.2.

12,19-Dihydroxyabieta-8,11,13-trien-7-one (15, 19-hydroxysugiol). 19-Hydroxyferruginol **14** (87 mg, 0.28 mmol) was dissolved in pyridine (2 mL) and Ac_2O (2 mL) was added. After stirring for 72 h, 25 mL of H_2O were added and the mixture was extracted with ethyl acetate (3 × 10 mL). The combined organic layers were washed with 6 % $NaHCO_3$ (2 × 10 mL), H_2O (2 × 10 mL), and brine (10 mL), dried and concentrated to afford the corresponding diacetate, 93 mg, as a pale oil which was used in the next step without further purification. The ^1H and ^{13}C NMR data were in agreement with those reported in the literature except in the assigned multiplicity in the ^{13}C NMR data for C1 and C4 (interchanged):[3] ^1H NMR (300 MHz) δ 6.94 (1H, s), 6.83 (1H, s), 4.31 (1H, d, J = 12), 3.99 (1H, d, J = 12), 2.30 (3H, s, OCO*Me*), 2.07 (3H, s, OCO*Me*), 1.20 (3H, s), 1.19 (3H, d, J = 6), 1.17 (3H, d, J = 6), 1.03 (3H, s); ^{13}C NMR (75 MHz) δ_C 171.3 (s), 169.9 (s), 148.0 (s), 146.2 (s), 136.9 (s), 132.6 (s), 126.9 (d), 118.1 (d), 66.8 (t), 50.9 (d), 38.6 (t), 37.4 (s), 37.0 (s), 35.8 (t), 30.4 (t), 27.2 (q), 27.1 (d), 25.6 (q), 23.0 (q), 22.9 (q), 20.9 (q), 20.9 (q), 19.1 (t), 18.8 (t).

The crude diacetate (90 mg) was dissolved in AcOH (2.5 mL) and cooled to 0-5 ºC. CrO_3 (50 mg, 0.47 mmol) was added and stirring continued for 20 h. Then, the mixture was poured into ice/H_2O (20 mL) and extracted with diethyl ether (3 × 10 mL). The combined organic extracts were washed with H_2O (2 × 10 mL), carefully saturated $NaHCO_3$ (2 × 10 mL), and brine (10 mL), dried and concentrated. The resulting residue was chromatographed on silica eluting with *n*-hexane-EtOAc (6:4) to give 80 mg (70 % overall yield, two steps) of the corresponding 7-oxo-diacetate as a colorless oil: ^{1}H NMR (300 MHz) δ 7.98 (1H, s), 6.99 (1H, s), 4.34 (1H, d, J = 10), 4.03 (1H, d, J = 10), 2.97 (1H, sept., J = 6), 2.83 (1H, dd, J = 16, 3), 2.68 (1H, dd, J = 16, 15), 2.32 (3H, s, OCO*Me*), 2.07 (3H, s, OCO*Me*), 1.27 (3H, s), 1.21 (3H, d, J = 6), 1.18 (3H, d, J = 6), 1.02 (3H, s); ^{13}C NMR (75 MHz) δ_C 197.6 (s), 171.1 (s), 169.0 (s), 154.5 (s), 152.7 (s), 138.6 (s), 128.5 (s), 126.4 (d), 118.0 (d), 66.6 (t), 49.5 (d), 37.9 (s), 37.8 (t), 36.7 (s), 36.0 (t), 36.0 (t), 27.2 (q), 26.7 (d), 23.6 (q), 22.7 (q), 22.6 (q), 20.9 (q), 20.9 (q), 18.4 (t).

The 7-oxo-diacetate of **14** (75 mg, 0.18 mmol) was dissolved in absolute MeOH (3 mL) and K_2CO_3 (140 mg, 1.0 mmol) was added. The reaction mixture was stirred for 20 h at rt and then diluted with H_2O (20 mL) and extracted with diethyl ether (3 × 10 mL). The combined organic extracts were dried and concentrated to afford 60 mg (66 % overall yield, three steps) of pure sugiol **15** as a colorless oil: $[\alpha]^{20}_D$ +18 (c 0.9, $CHCl_3$). ^{1}H NMR (300 MHz) δ 7.90 (1H, s), 6.76 (1H, s), 3.88 (1H, d, J = 12), 3.62 (1H, d, J = 12), 3.18 (1H, sept., J = 6), 2.80-2.60 (2H, m), 2.22-2.18 (1H, m), 1.98 (1H, dd, J = 12, 3), 1.94-1.89 (1H, m), 1.70-1.50 (3H, m), 1.23 (3H, d, J = 6), 1.22 (3H, d, J = 6), 1.19 (3H, s), 1.01 (3H, s); ^{13}C NMR (75 MHz) δ_C 198.8 (s), 159.4 (s), 156.4 (s), 133.3 (s), 126.6 (d), 123.6 (s), 110.0 (d), 64.9 (t), 49.8 (d), 38.2 (s), 37.9 (t), 37.8 (s), 35.8 (t), 35.1 (t), 26.7 (d), 26.3 (q), 23.7 (q), 22.4 (q), 22.2 (q), 18.5 (t); HRMS (ESI) *m/z* 317.2122 $[M+H]^+$, calcd for $C_{20}H_{29}O_3$: 317.2117; Anal. calcd. for $C_{20}H_{28}O_3$: C, 75.9; H, 8.9. Found: C, 75.5; H, 9.1.

Methyl 12-hydroxy-7-oxoabieta-8,11,13-trien-19-oate (16, methyl 12-hydroxy-7-oxocallitrisate). The acetate **11** (250 mg, 0.64 mmol) was dissolved in absolute MeOH (9 mL) and K_2CO_3 (450 mg, 3.24 mmol) was added in portions at rt. The reaction mixture was stirred for 1 h at rt and then diluted with H_2O (60 mL) and extracted with diethyl ether (3 × 15 mL). The combined organic extracts were dried and concentrated to afford 220 mg (>99 %) of pure sugiol **16** as a white solid: mp 252-256 ºC; $[\alpha]^{20}_D$ +84 (c 1.1, $CHCl_3$). ^{1}H NMR (300 MHz) δ 7.94 (1H, s), 6.78 (1H, s), 3.69 (3H, s), 3.23-3.12 (2H, m), 2.93 (1H, dd, J = 18, 3), 2.31-2.20 (2H, m), 2.03 (1H, dd, J = 15, 3), 2.00-1.96 (1H, m), 1.69-1.63 (1H, m), 1.50 (1H, ddd, J = 12, 12, 3), 1.25 (3H, d, J = 6), 1.24 (3H, s), 1.24 (3H, d, J = 6), 1.12-1.08 (1H, m), 1.07 (3H, s); ^{13}C NMR (75 MHz) δ_C 198.5 (s), 177.2 (s), 159.0 (s), 154.8 (s), 133.4 (s), 126.4 (d), 123.9 (s), 110.9 (d), 51.6 (q), 50.3 (d), 43.9 (s), 38.4 (t), 38.3 (s), 37.4 (t), 37.4 (t), 27.9 (q), 26.8 (d), 22.4 (q), 22.3 (q), 21.2 (q), 19.6 (t); HRMS (ESI) *m/z* 345.2071 [M+ H]$^+$, calcd for $C_{21}H_{29}O_4$: 345.2066; Anal. calcd. for $C_{21}H_{28}O_4$: C, 73.2; H, 8.2. Found: C, 73.2; H, 8.4.

12-Hydroxy-7-oxoabieta-8,11,13-trien-19-oic acid (8a, 4-epi-liquiditerpenoic acid A). A suspension of ester **16** (125 mg, 0.36 mmol) and LiI (340 mg, 2.54 mmol) in 2,4,6-collidine (2 mL) was heated at reflux for 2 h under an Argon atmosphere and then cooled to rt. The mixture was treated with ice and 6 N HCl (20 mL) carefully and extracted with DCM (3 × 10 mL). The organic extracts were washed with brine, dried and concentrated. The resulting brown residue (113 mg) was dissolved in DCM and chromatographed on silica eluting with *n*-hexane-EtOAc (4:6) to give 20 mg (17%) (for some reason the compound seems to be retained on silica and do not come out with more polar eluents) of acid **8a** as a light pale solid: mp 166-168 ºC; $[\alpha]^{20}_D$ +60 (c 0.5, $CHCl_3$). ^{1}H NMR (300 MHz, CD_3OD) δ 7.82 (1H, s), 6.80 (1H, s), 3.28-3.18 (2H, m), 2.83 (1H, dd, J = 18, 3), 2.33-2.23 (2H, m), 2.15-2.07 (1H, m), 2.03 (1H, dd, J = 15, 3), 1.70-1.63 (1H, m),

1.52 (1H, ddd, J = 12, 12, 3), 1.27 (3H, s), 1.22-1.19 (2H,m), 1.21 (3H, d, J = 6), 1.20 (3H, d, J = 6), 1.19 (3H, s); ^{13}C NMR (75 MHz, CD_3OD) δ_C 200.6 (s), 180.4 (s), 162.4 (s), 156.8 (s), 135.0 (s), 126.8 (d), 123.9 (s), 111.3 (d), 51.6 (d), 44.7 (s), 39.6 (t), 38.5 (t), 38.4 (t), 28.6 (q), 27.9 (d), 22.8 (q), 22.7 (q), 22.0 (q), 20.8 (t); HRMS (ESI) *m/z* 331.1916 $[M+ H]^+$, calcd for $C_{20}H_{27}O_4$: 331.1909; Anal. calcd. for $C_{20}H_{26}O_4$: C, 72.7; H, 7.9. Found: C, 72.4; H, 8.1.

References

(1) Alvarez-Manzaneda, E. J.; Chahboun, R.; Cabrera, E.; Alvarez, E.; Alvarez-Manzaneda, R.; Lachkar, M.; Messouri, I. Synthesis of Phenol Abietane Diterpenes Based on the Oxidative Radical Cyclization Utilizing the $Mn(OAc)_3/Ac_2O$ System. *Synlett.* **2007**, *15*, 2425-2429.
(2) Campello, J. P.; Fonseca, S. F.; Chang, C.-J.; Wenkert, E. Terpenes of *Podocarpus lambertius*. *Phytochem.* **1975**, *14*, 243-248.
(3) Cambie, R. C.; Cox, R. E.; Sidwell, D.; Phenolic diterpenoids of *Podocarpus ferrugineus* and other Podocarps. *Phytochem.* **1984**, *23*, 333-336.
(4) Moujir, L.; Seca, A. M. L.; Silva, A. M. S.; Barreto, M. C. Cytotoxic activity of diterpenes and extracts of *Juniperus brevifolia*. *Planta Med.* **2008**, *74*, 751-753.

Compound 12

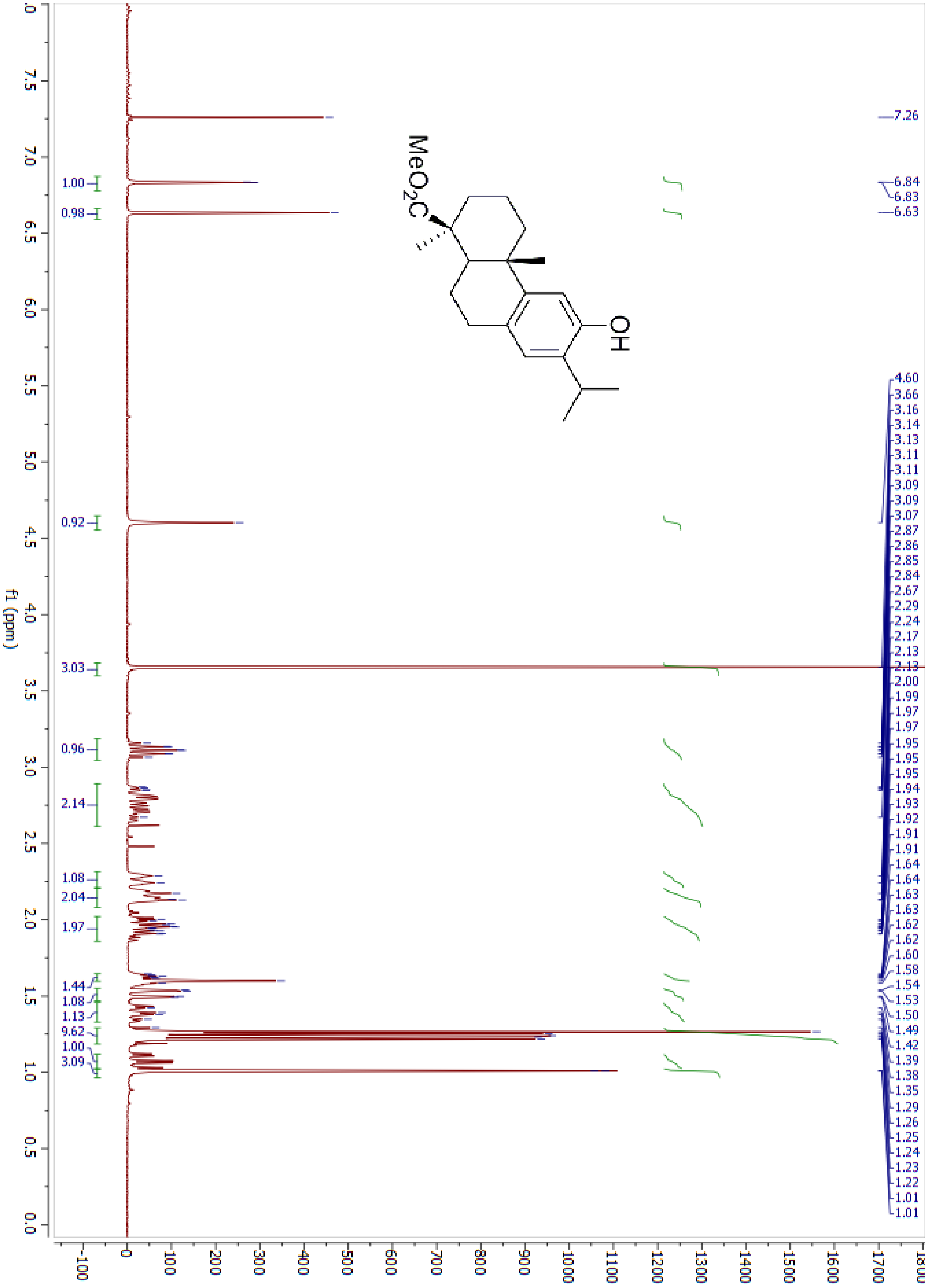

Compound 12

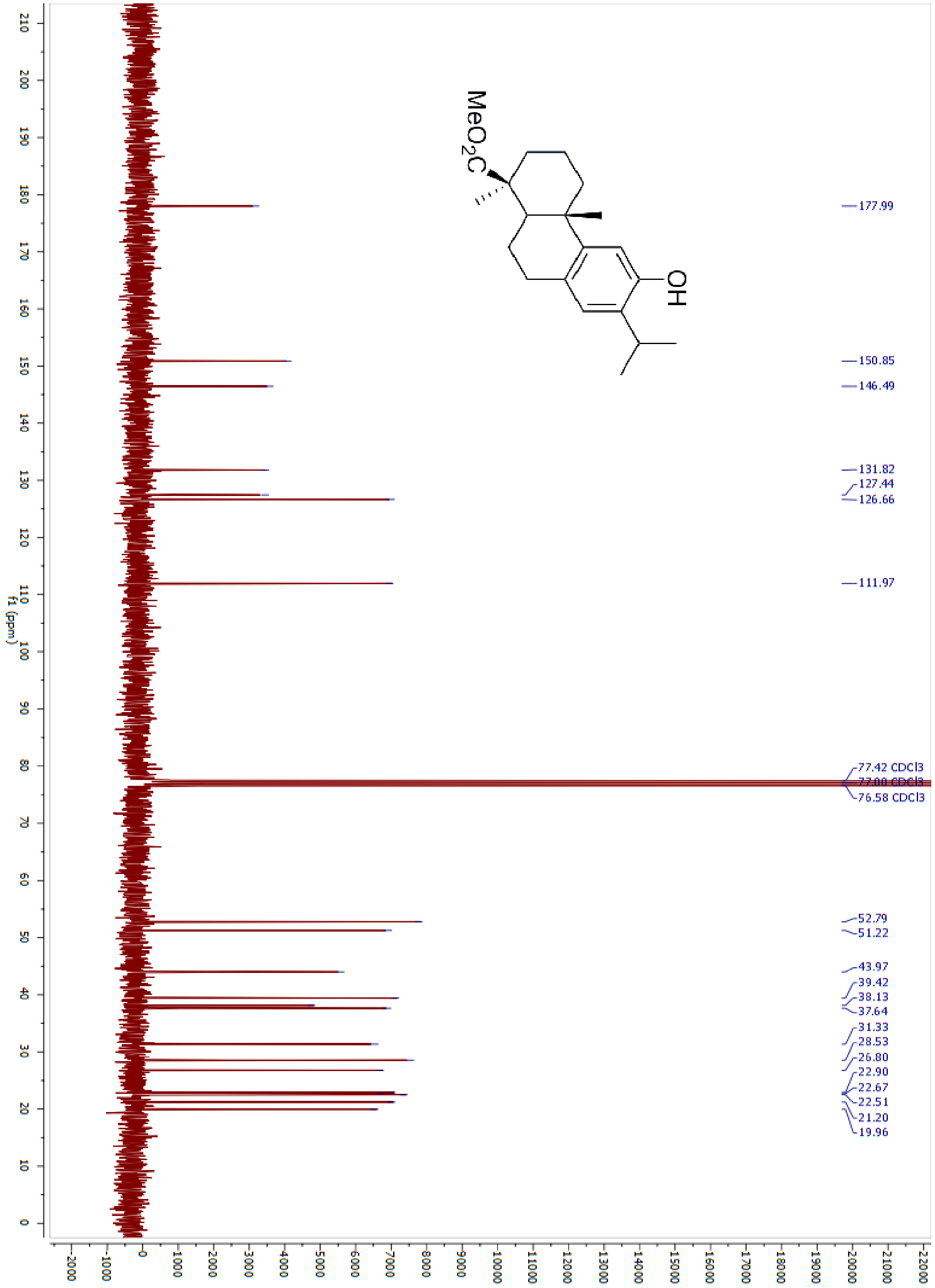

MeO2C
OH
177.99
150.85
146.49
131.82
127.44
126.66
111.97
77.42 CDCl3
77.00 CDCl3
76.58 CDCl3
52.79
51.22
43.97
39.42
38.13
37.64
31.33
28.53
26.80
22.90
22.67
22.51
21.20
19.96
f1 (ppm)

Compound 12

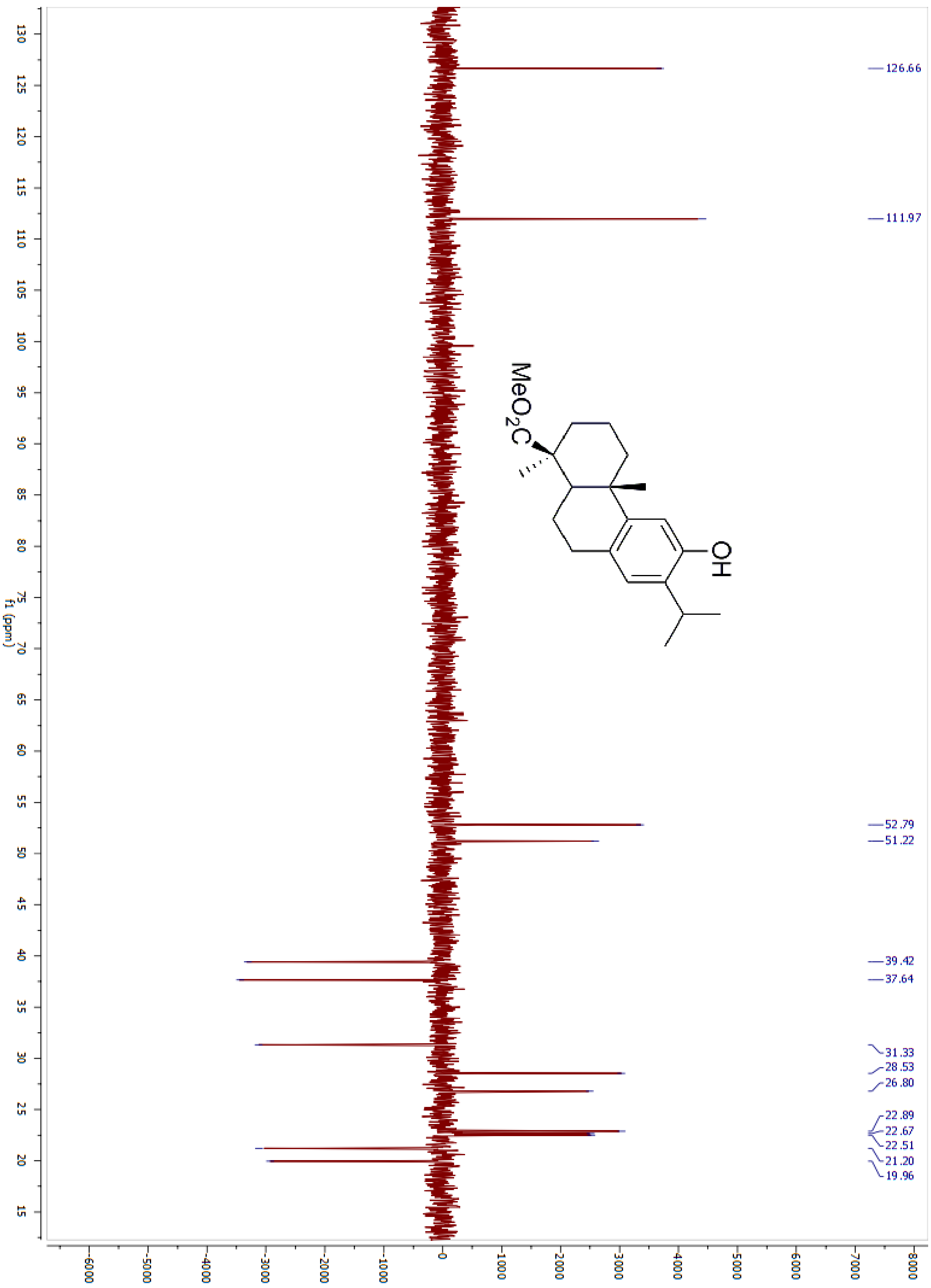
126.66
111.97
52.79
51.22
39.42
37.64
31.33
28.53
26.80
22.89
22.67
22.51
21.20
19.96
f1 (ppm)
MeO2C
OH

Compound 13

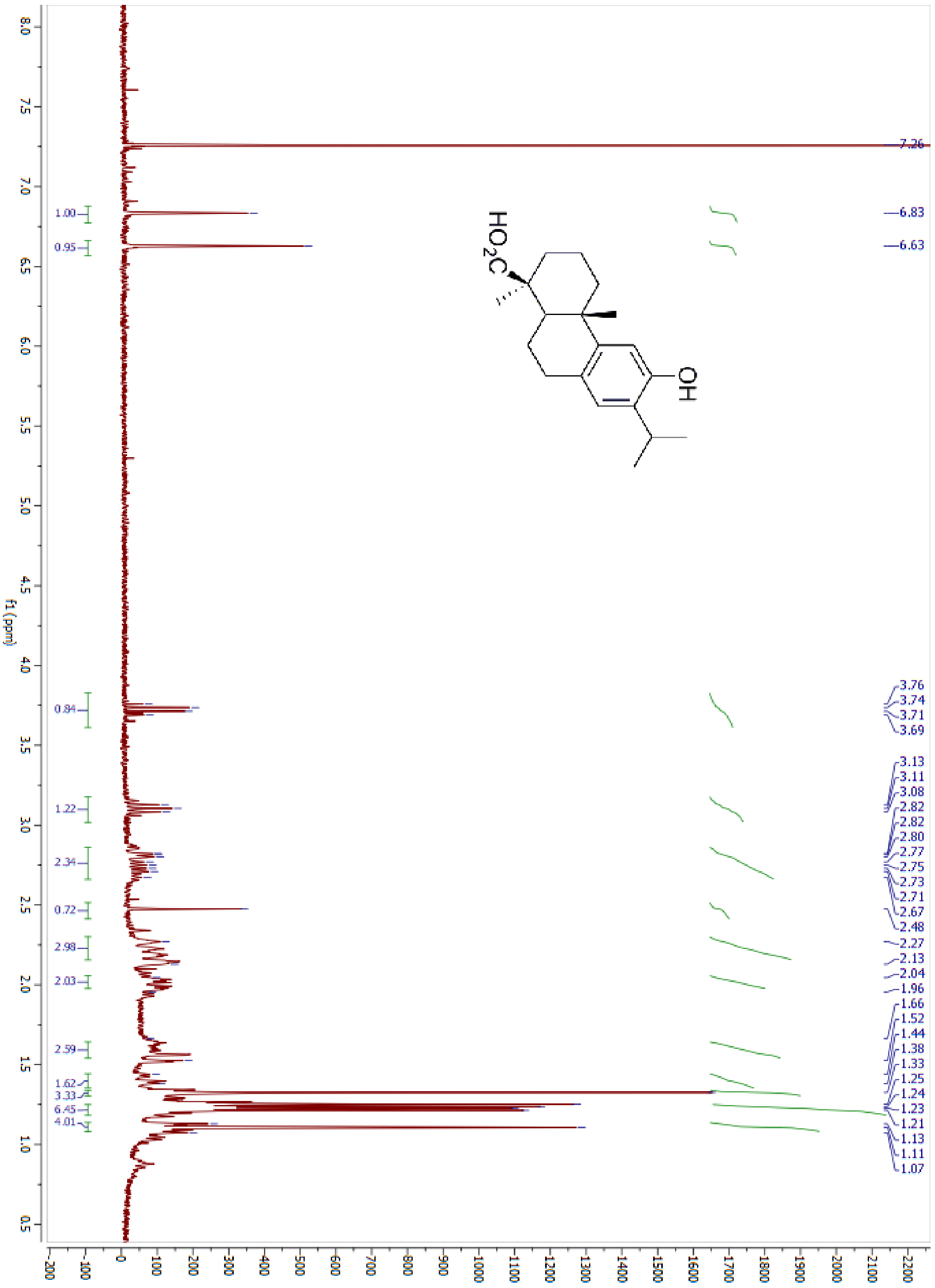

Compound 13

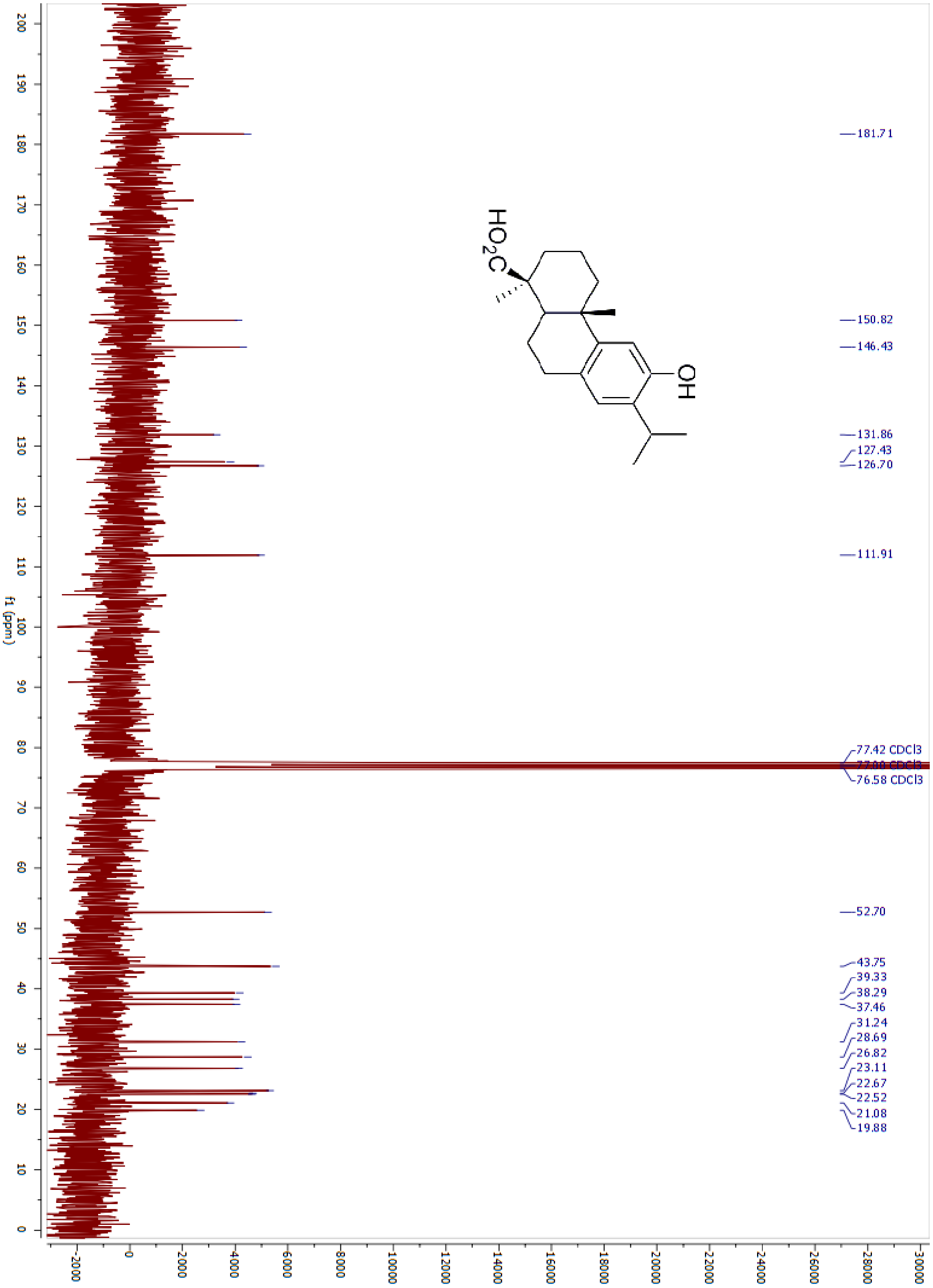

HO2C
OH
181.71
150.82
146.43
131.86
127.43
126.70
111.91
77.42 CDCl3
76.58 CDCl3
52.70
43.75
39.33
38.29
37.46
31.24
28.69
26.82
23.11
22.67
22.52
21.08
19.88
f1 (ppm)

Compound 13

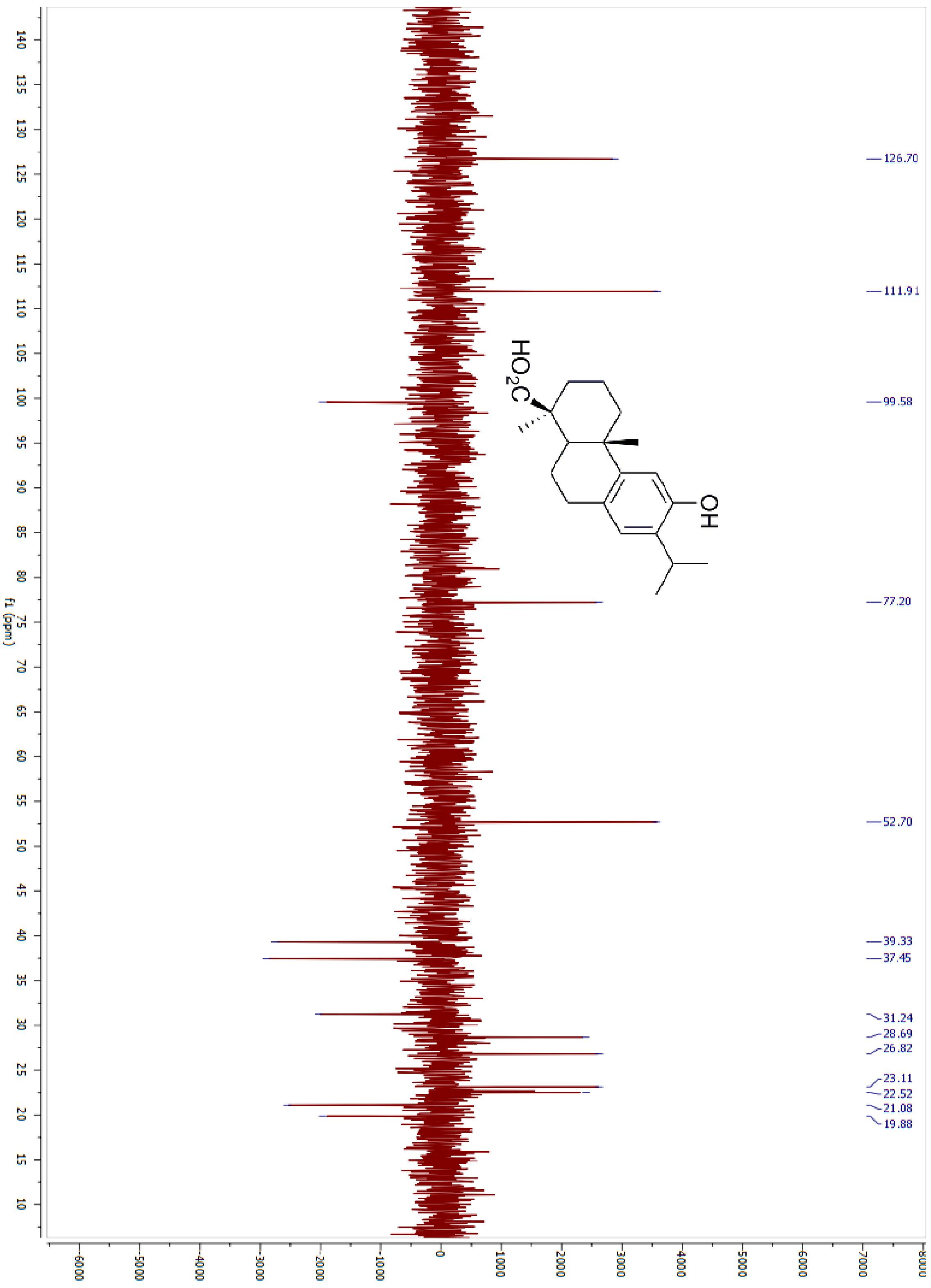

126.70
111.91
99.58
77.20
52.70
39.33
37.45
31.24
28.69
26.82
23.11
22.52
21.08
19.88
f1 (ppm)
HO2C
OH

Compound 14

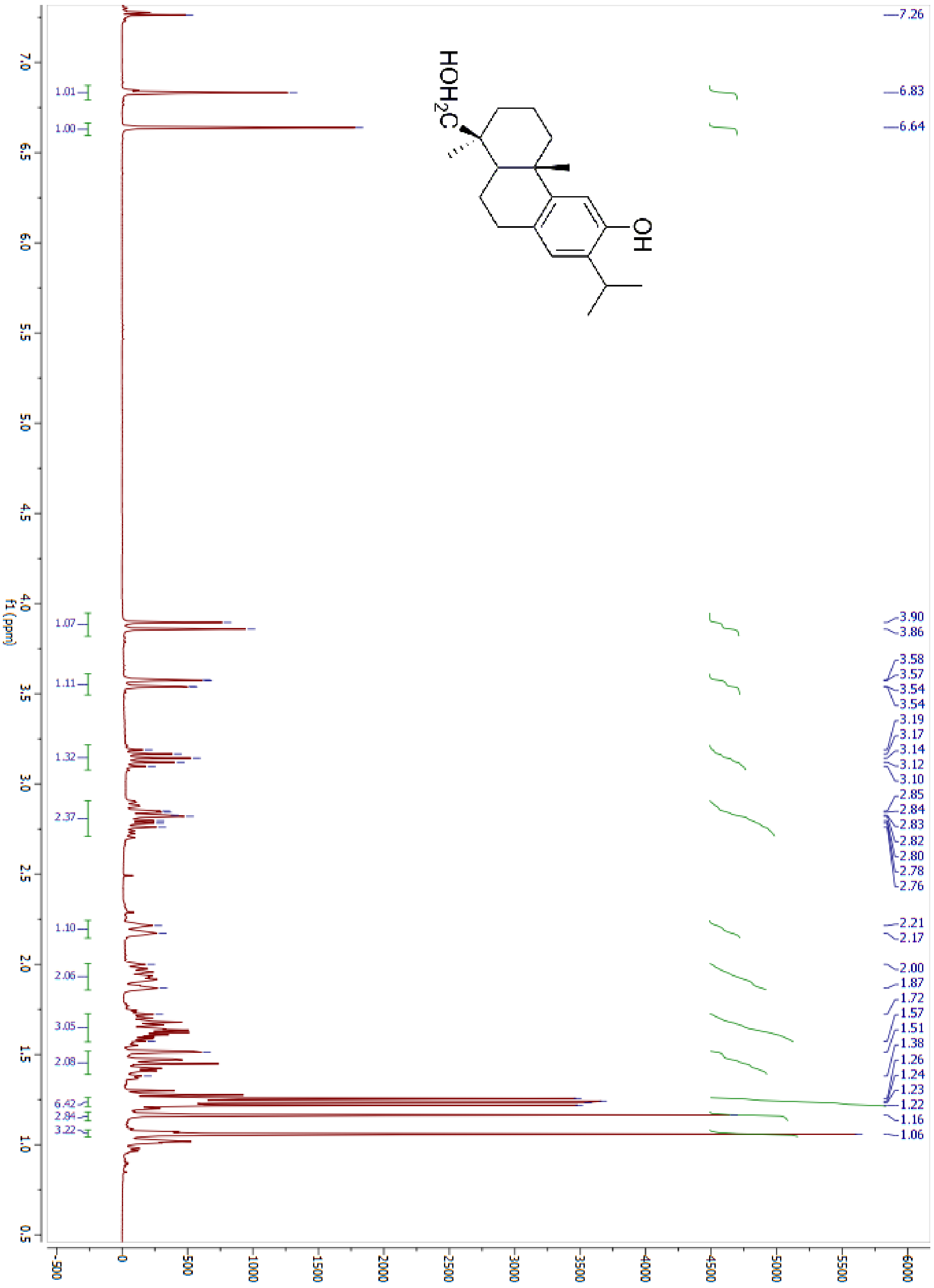

HOH2C
OH
7.26
6.83
6.64
3.90
3.86
3.58
3.57
3.54
3.54
3.19
3.17
3.14
3.12
3.10
2.85
2.84
2.83
2.82
2.80
2.78
2.76
2.21
2.17
2.00
1.87
1.72
1.57
1.51
1.38
1.26
1.24
1.23
1.22
1.16
1.06
1.01
1.00
1.07
1.11
1.32
2.37
1.10
2.06
3.05
2.08
6.42
2.84
3.22
f1 (ppm)

Compound 14

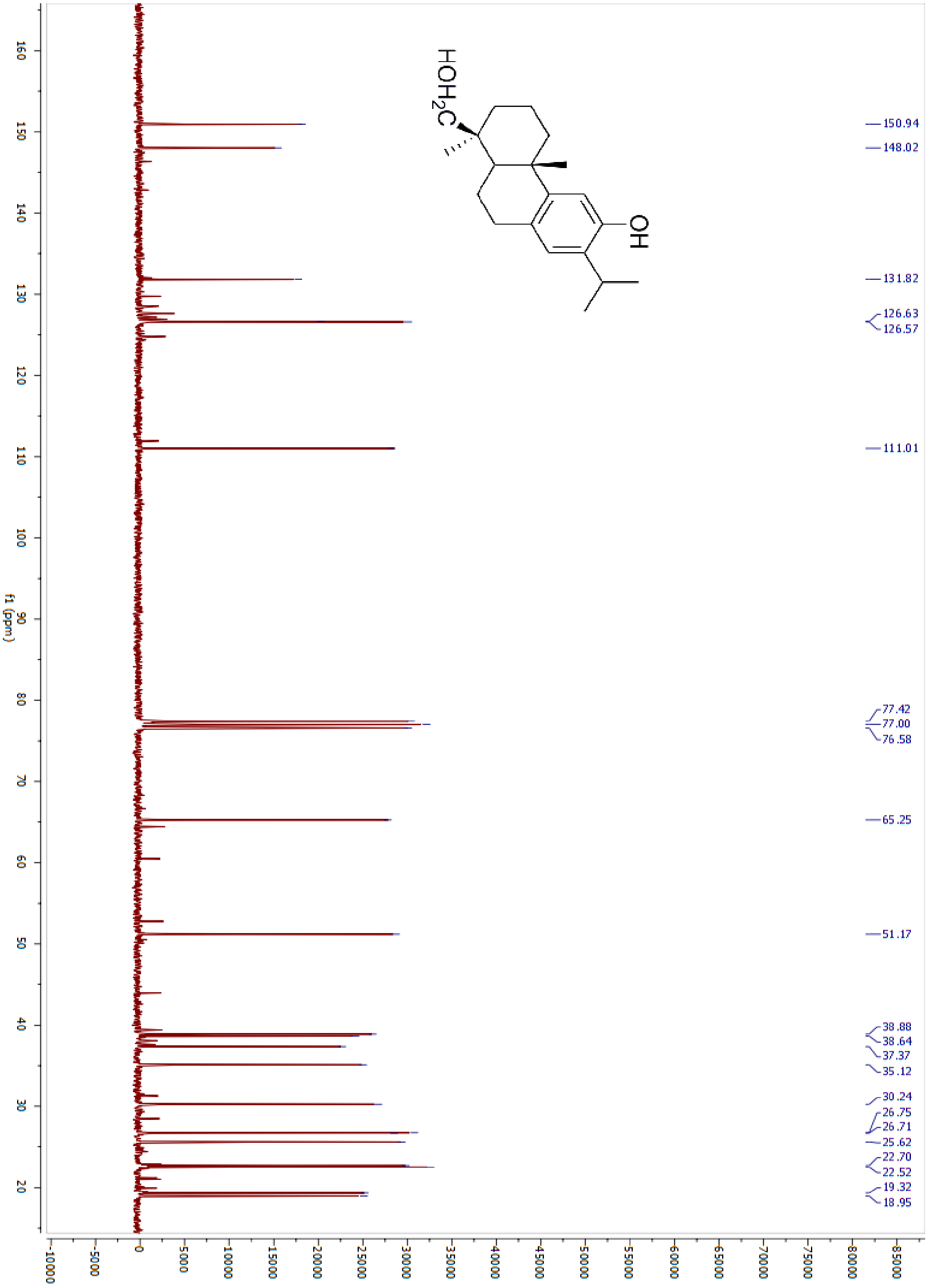

150.94
148.02
131.82
126.63
126.57
111.01
77.42
77.00
76.58
65.25
51.17
38.88
38.64
37.37
35.12
30.24
26.75
26.71
25.62
22.70
22.52
19.32
18.95
f1 (ppm)
HOH2C
OH

Compound 14

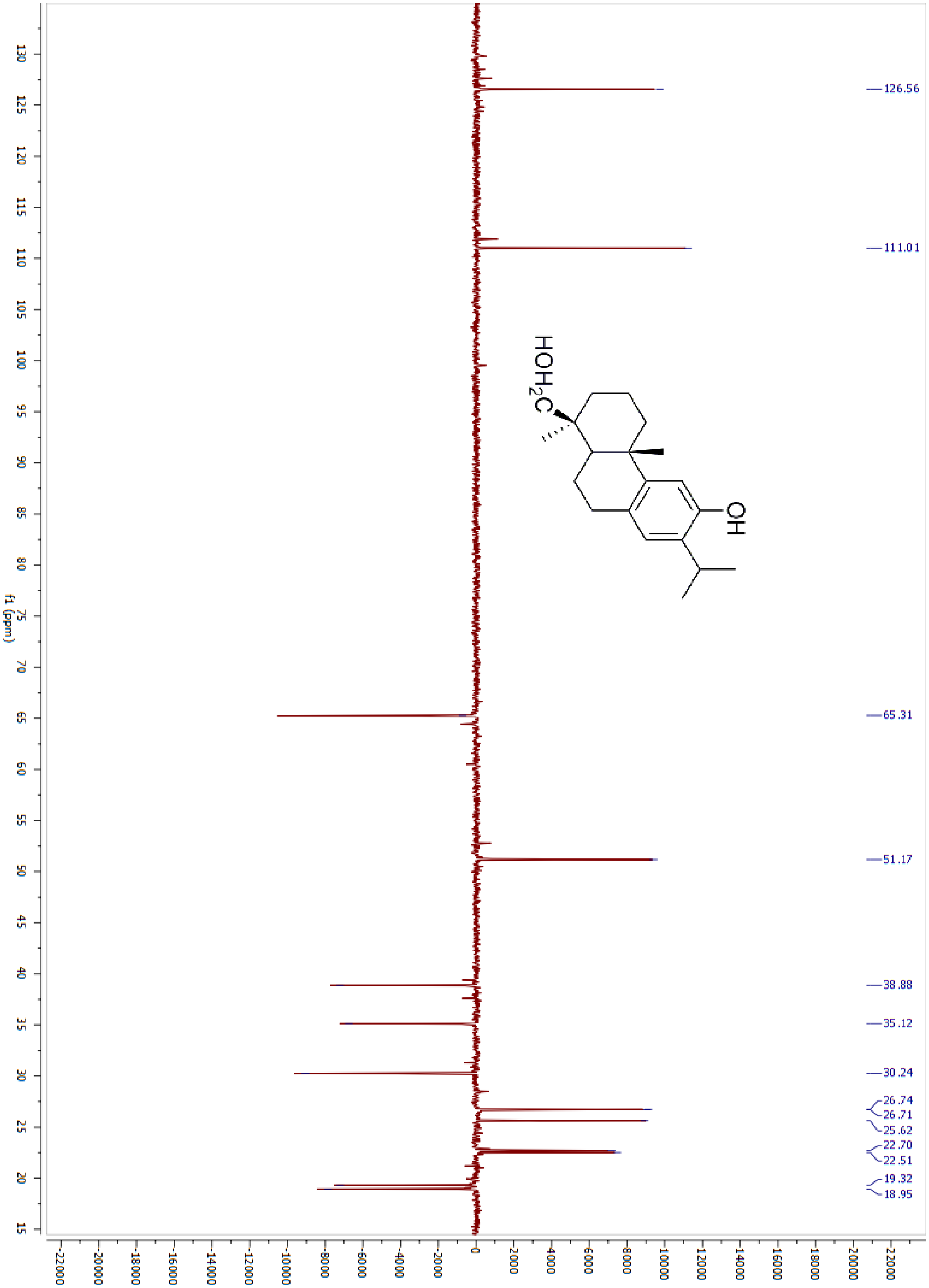
126.56
111.01
65.31
51.17
38.88
35.12
30.24
26.74
26.71
25.62
22.70
22.51
19.32
18.95
HOH2C
OH
f1 (ppm)

Compound 15

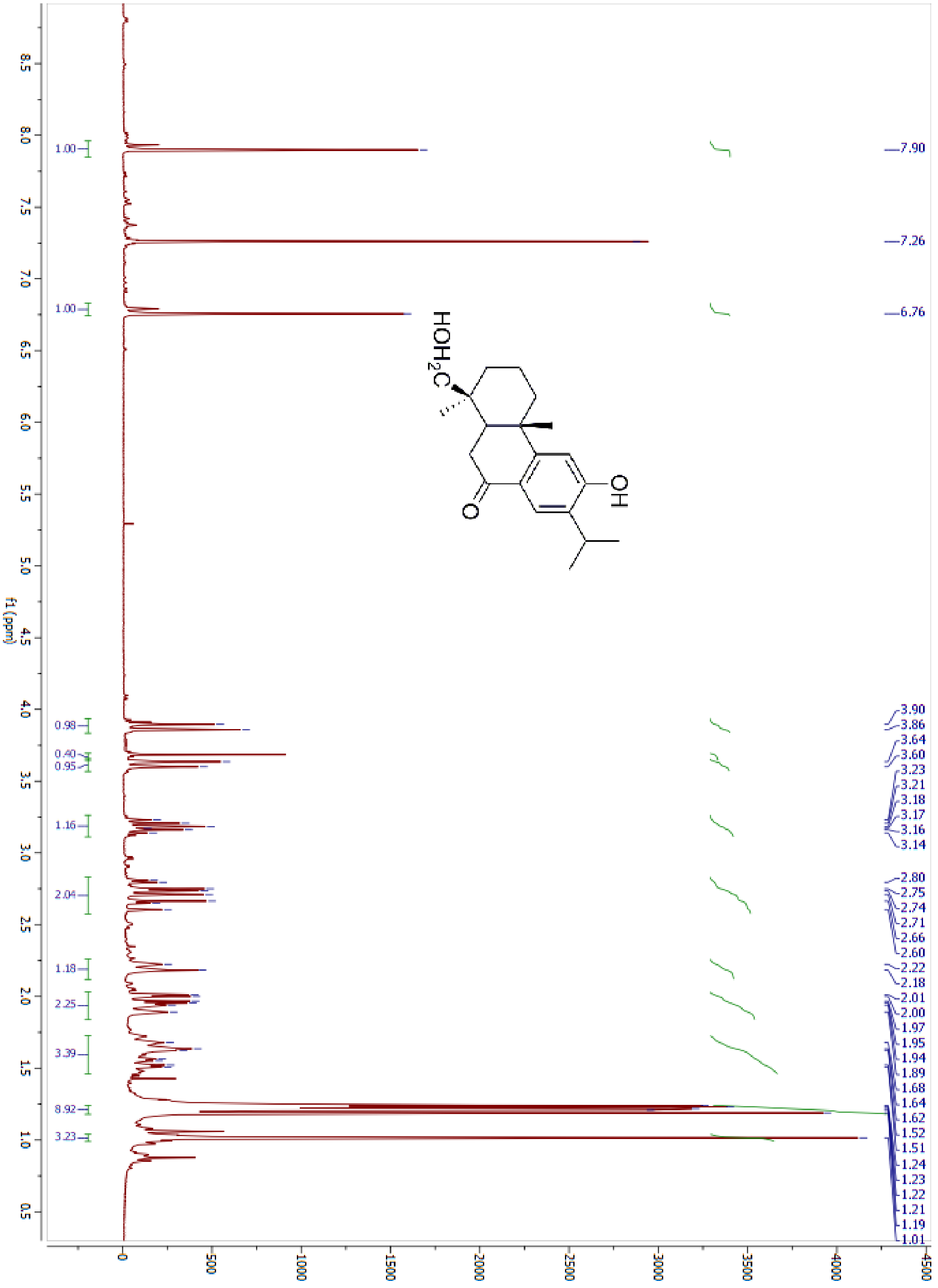
f1 (ppm)
HOH2C
OH
O
7.90
7.26
6.76
3.90
3.86
3.64
3.60
3.23
3.21
3.18
3.17
3.16
3.14
2.80
2.75
2.74
2.71
2.66
2.60
2.22
2.18
2.01
2.00
1.97
1.95
1.94
1.89
1.68
1.64
1.62
1.52
1.51
1.24
1.23
1.22
1.21
1.19
1.01
1.00
1.00
0.98
0.40
0.95
1.16
2.04
1.18
2.25
3.39
8.92
3.23

Compound 15

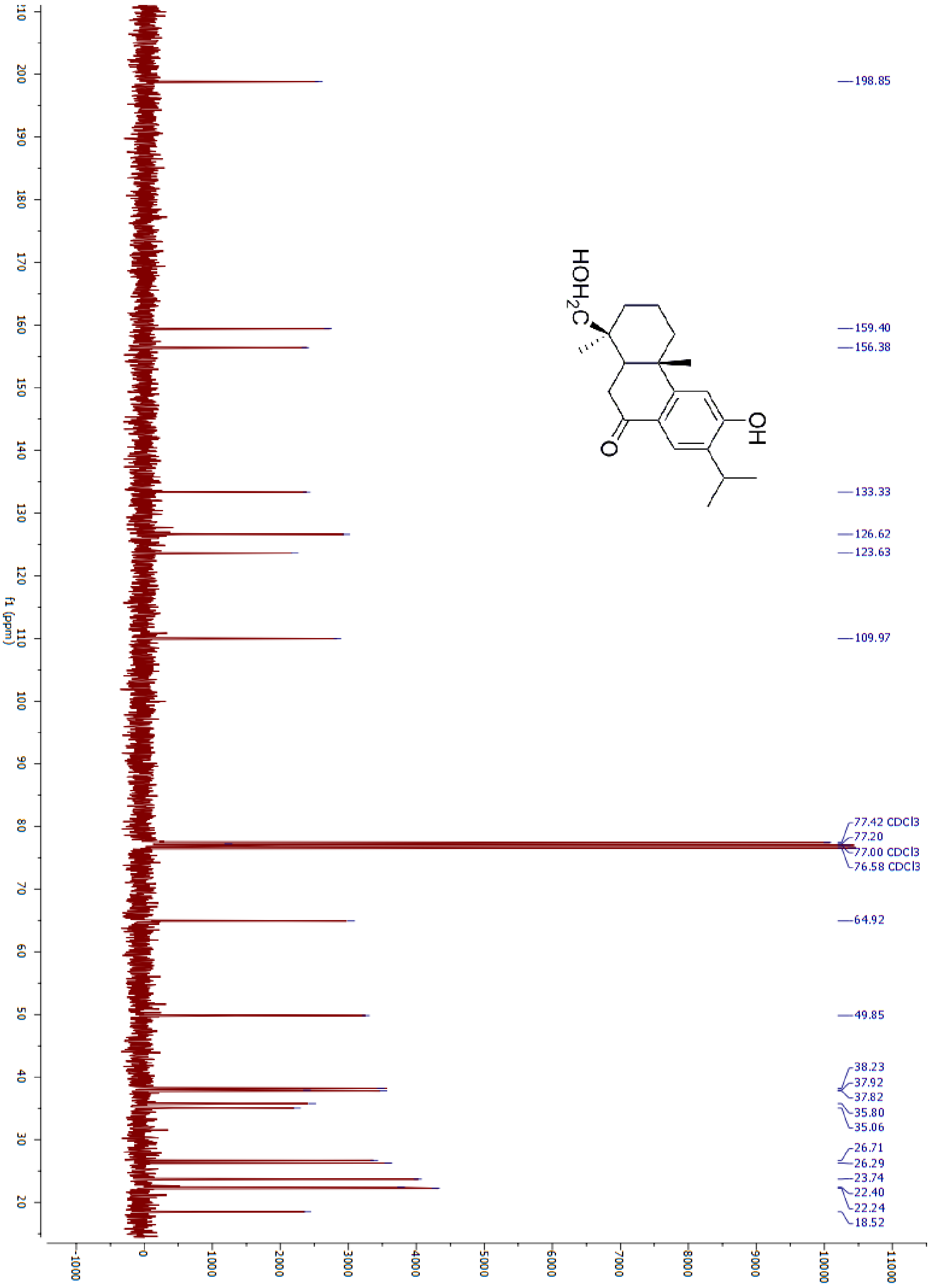

198.85
159.40
156.38
133.33
126.62
123.63
109.97
77.42 CDCl3
77.20
77.00 CDCl3
76.58 CDCl3
64.92
49.85
38.23
37.92
37.82
35.80
35.06
26.71
26.29
23.74
22.40
22.24
18.52
f1 (ppm)
HOH2C
OH
O

Compound 15

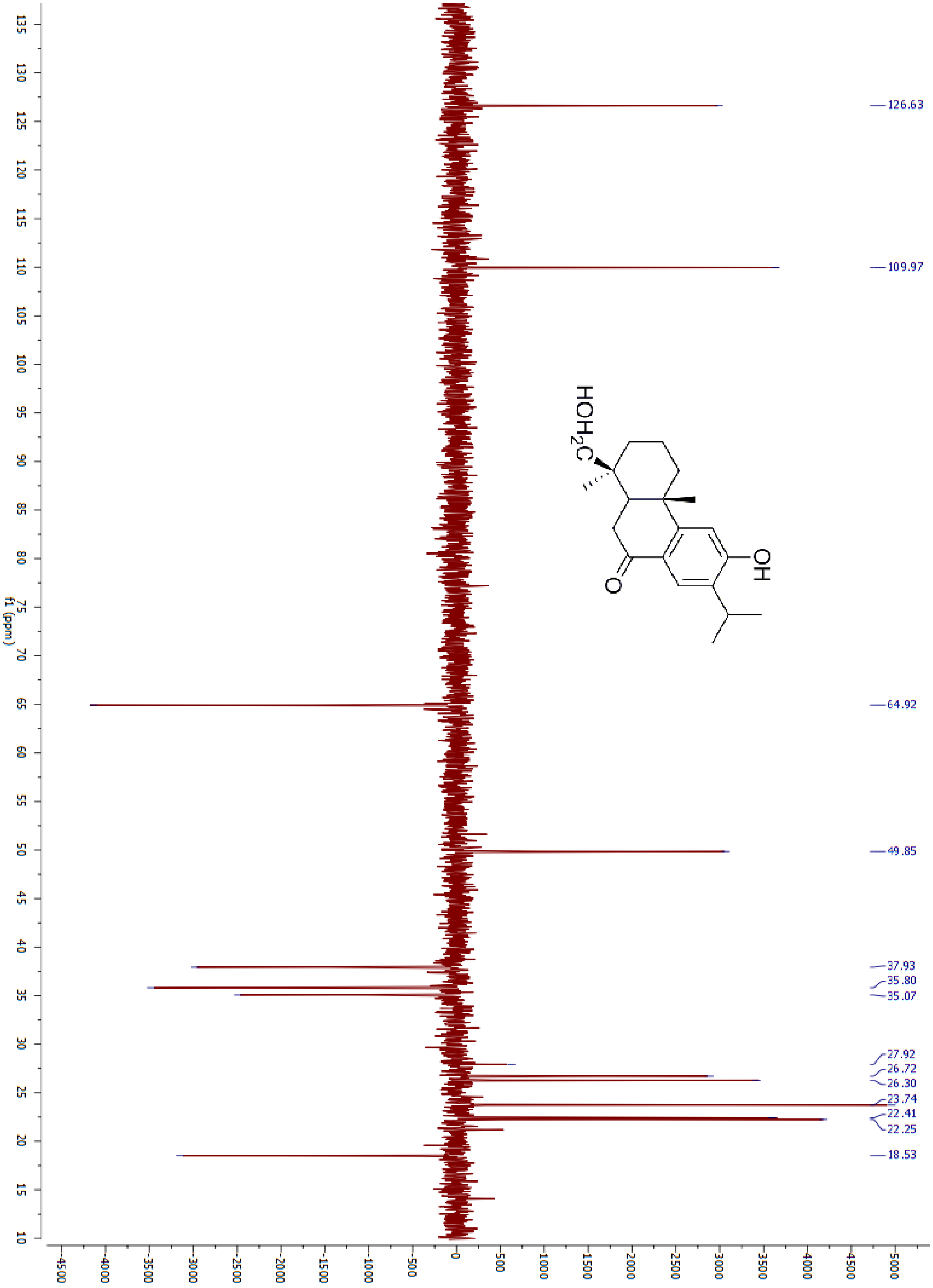
126.63
109.97
64.92
49.85
37.93
35.80
35.07
27.92
26.72
26.30
23.74
22.41
22.25
18.53
f1 (ppm)
HOH2C
OH
O

Compound 16

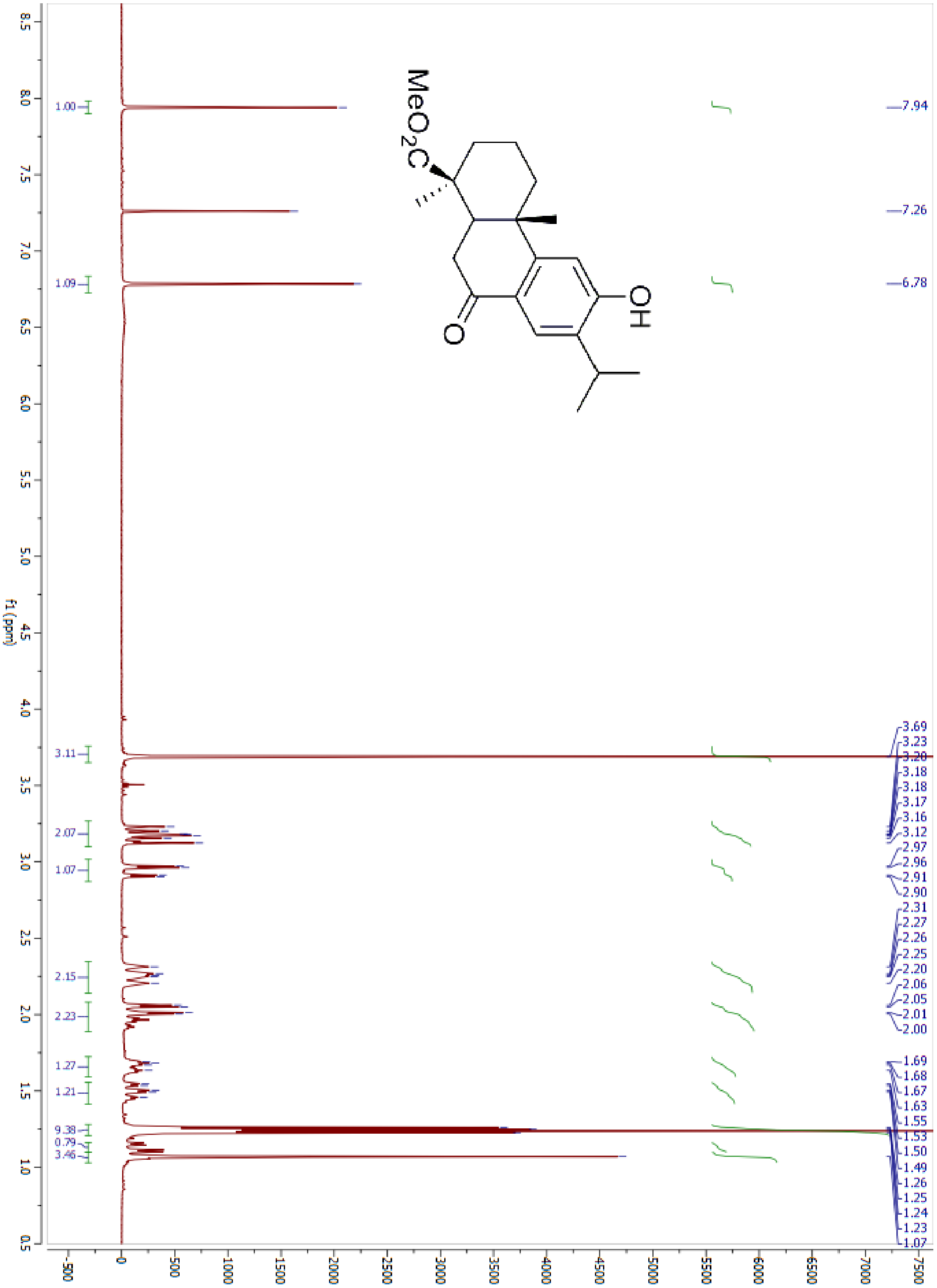

Compound 16

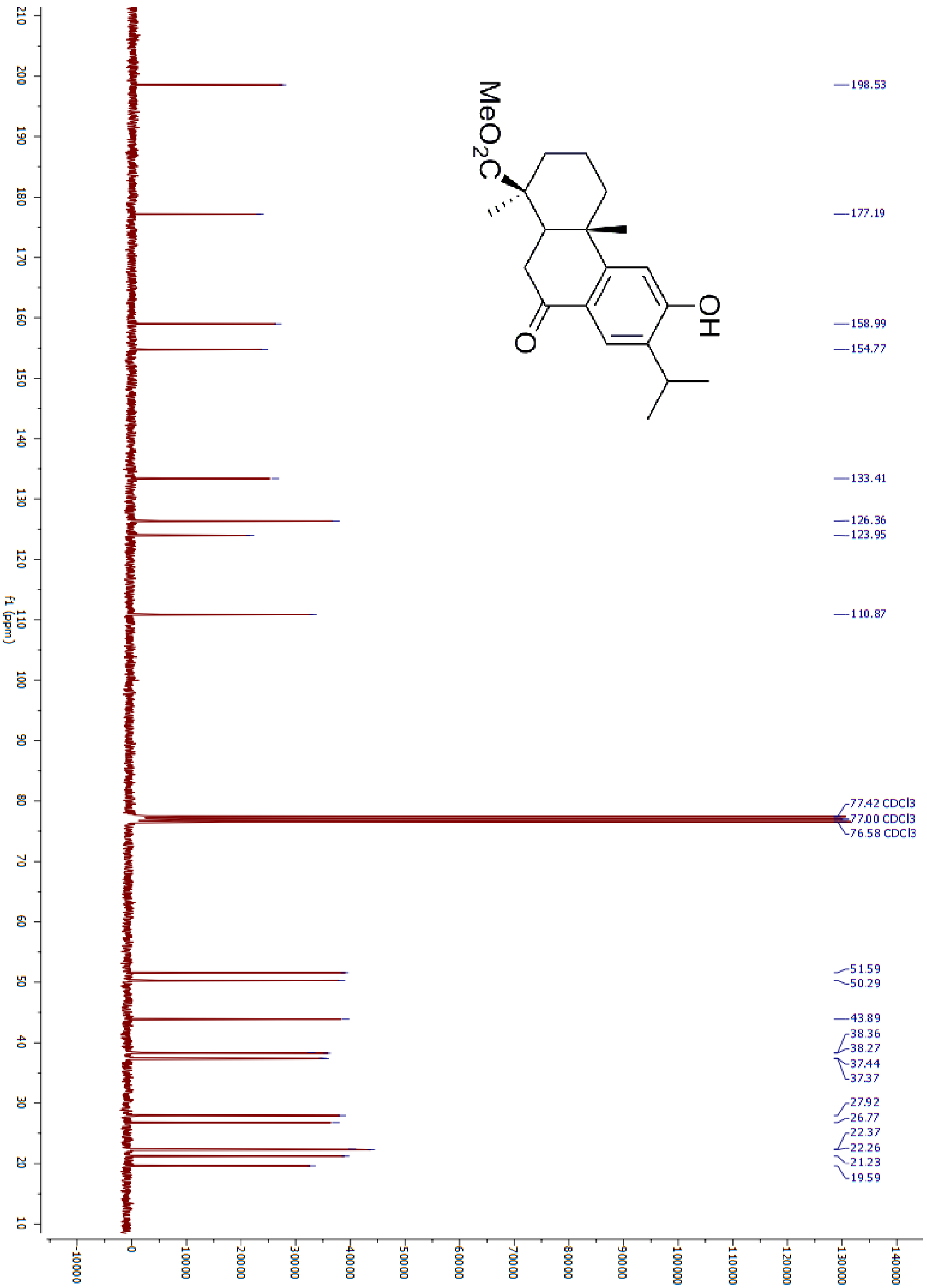
MeO2C
OH
O
198.53
177.19
158.99
154.77
133.41
126.36
123.95
110.87
77.42 CDCl3
77.00 CDCl3
76.58 CDCl3
51.59
50.29
43.89
38.36
38.27
37.44
37.37
27.92
26.77
22.37
22.26
21.23
19.59
f1 (ppm)

Compound 16

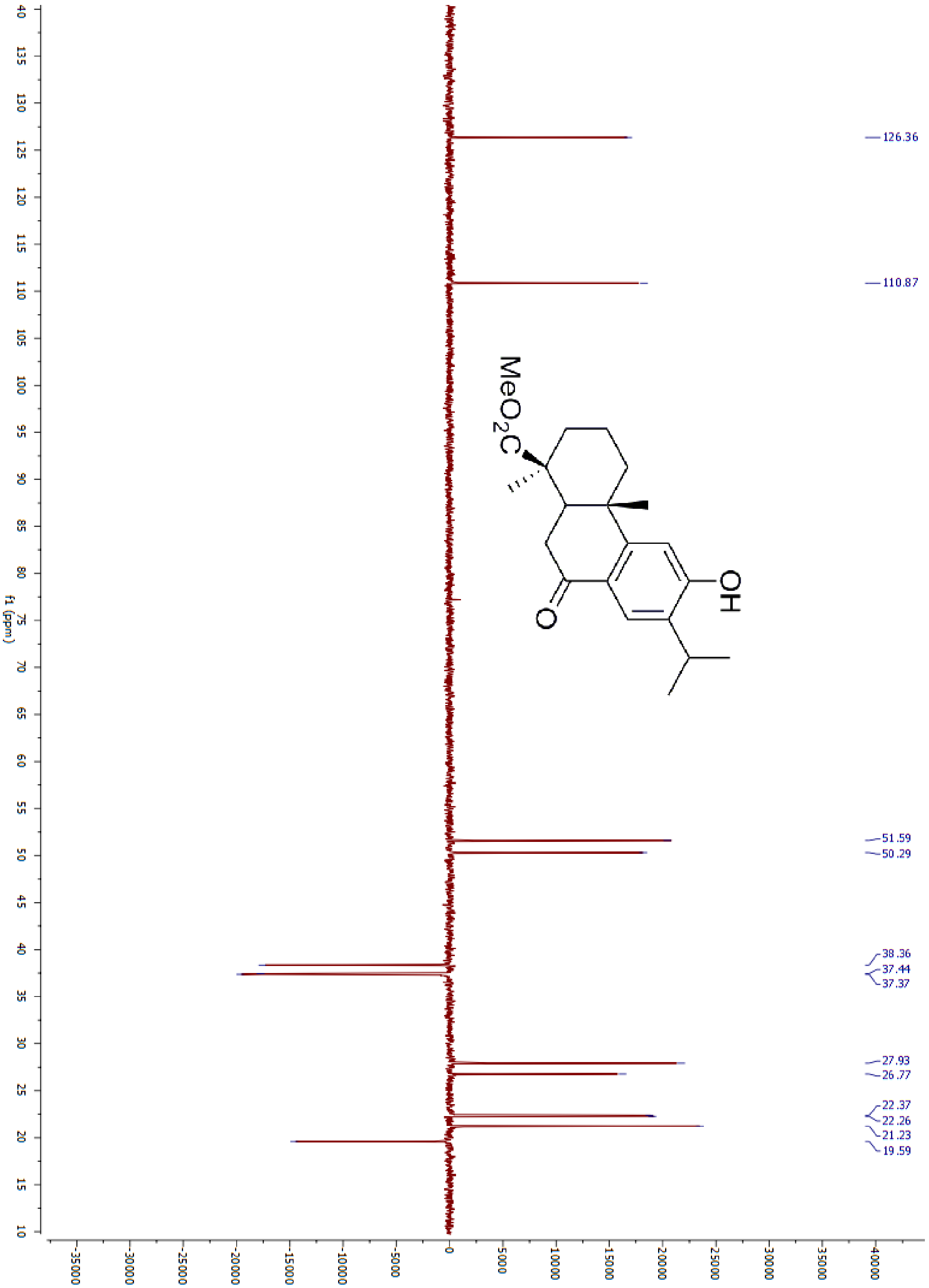
126.36
110.87
51.59
50.29
38.36
37.44
37.37
27.93
26.77
22.37
22.26
21.23
19.59
f1 (ppm)
MeO2C
OH
O

Compound 8a

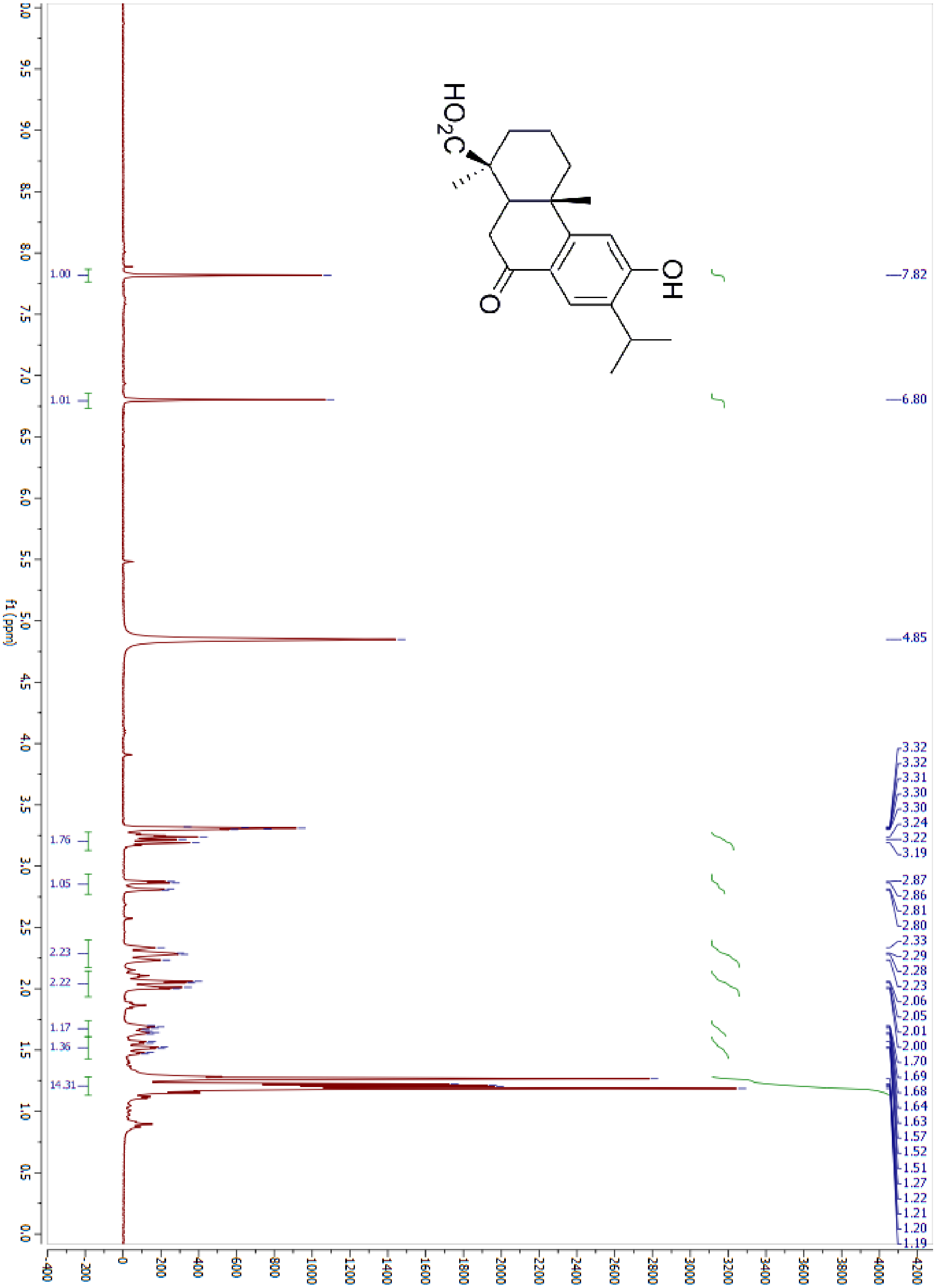

HO2C
OH
O
f1 (ppm)
1.00
1.01
1.76
1.05
2.23
2.22
1.17
1.36
14.31
7.82
6.80
4.85
3.32
3.32
3.31
3.30
3.30
3.24
3.22
3.19
2.87
2.86
2.81
2.80
2.33
2.29
2.28
2.23
2.06
2.05
2.01
2.00
1.70
1.69
1.68
1.64
1.63
1.57
1.52
1.51
1.27
1.22
1.21
1.20
1.19

Compound 8a

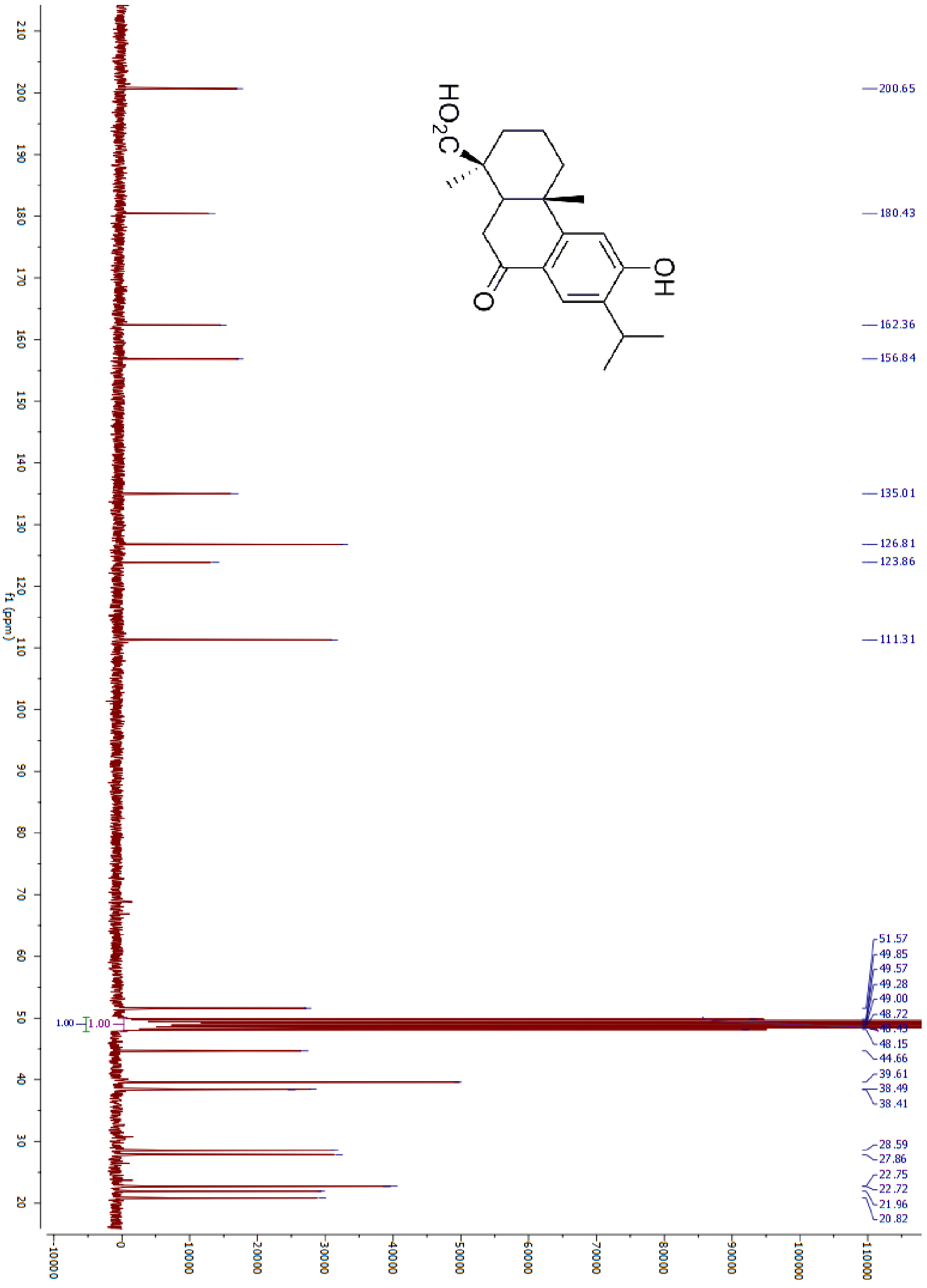

HO2C
OH
O
200.65
180.43
162.36
156.84
135.01
126.81
123.86
111.31
51.57
49.85
49.57
49.28
49.00
48.72
48.15
44.66
39.61
38.49
38.41
28.59
27.86
22.75
22.72
21.96
20.82
1.00
f1 (ppm)

Compound 8a

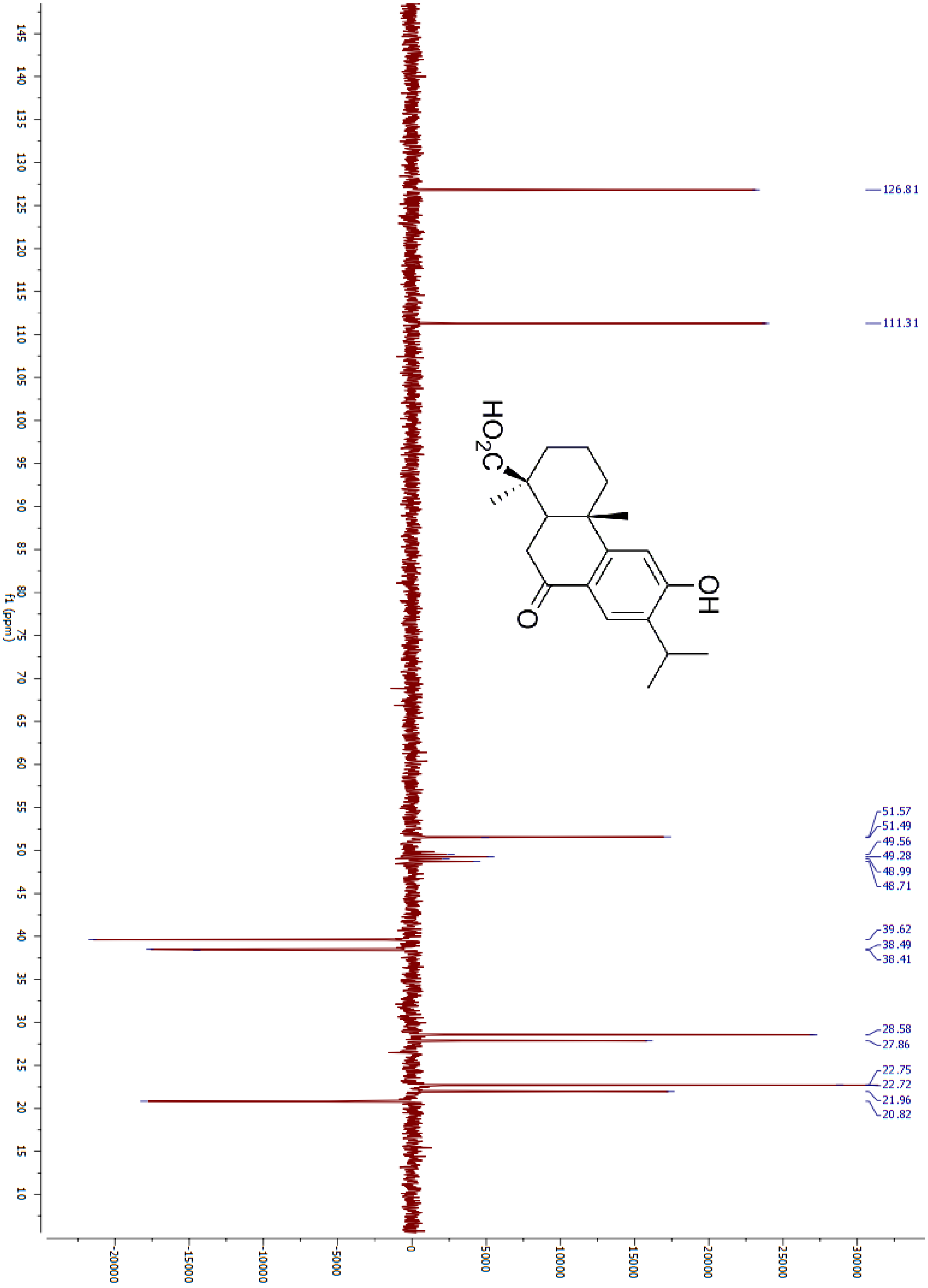
126.81
111.31
51.57
51.49
49.56
49.28
48.99
48.71
39.62
38.49
38.41
28.58
27.86
22.75
22.72
21.96
20.82
f1 (ppm)
HO2C
OH
O
